# Cargo regulates clathrin-coated pit invagination via clathrin light chain phosphorylation

**DOI:** 10.1083/jcb.201805005

**Published:** 2018-12-03

**Authors:** Hannes Maib, Filipe Ferreira, Stéphane Vassilopoulos, Elizabeth Smythe

**Affiliations:** 1 Centre for Membrane Interactions and Dynamics, Department of Biomedical Science, University of Sheffield, Sheffield, UK; 2 Sorbonne Université, INSERM, Institute of Myology, Centre for Research in Myology, UMRS 974, Paris, France

## Abstract

Clathrin light chains (CLCs) control selective uptake of a range of G protein–coupled receptors (GPCRs), although the mechanism by which this occurs has remained elusive thus far. In particular, site-specific phosphorylation of CLCb controls the uptake of the purinergic GPCR P2Y_12_, but it is dispensable for the constitutive uptake of the transferrin receptor (TfR). We demonstrate that phosphorylation of CLCb is required for the maturation of clathrin-coated pits (CCPs) through the transition of flat lattices into invaginated buds. This transition is dependent on efficient clathrin exchange regulated by CLCb phosphorylation and mediated through auxilin. Strikingly, this rearrangement is required for the uptake of P2Y_12_ but not TfR. These findings link auxilin-mediated clathrin exchange to early stages of CCP invagination in a cargo-specific manner. This supports a model in which CCPs invaginate with variable modes of curvature depending on the cargo they incorporate.

## Abstract (precis)

Phosphorylation of clathrin light chains (CLCs) regulates GPCR uptake but is dispensable for transferrin internalization. Maib et al. show that CLCb phosphorylation is required for efficient auxilin-mediated clathrin exchange to promote coated pit invagination in a cargo-specific manner.

## Introduction

Clathrin-mediated endocytosis (CME) is a major pathway that controls receptor uptake from the plasma membrane and is essential for physiological processes including synaptic vesicle recycling, developmental signaling, and immune responses (McMahon and Boucrot, 2011; Mettlen et al., 2018). A triskelion is composed of three units of clathrin heavy chain, each associated with a clathrin light chain (CLC; Brodsky, 2012; Maib et al., 2017). Clathrin triskelia assemble onto the plasma membrane to form clathrin-coated pits (CCPs), which trap receptor cargoes and invaginate, becoming spherical clathrin-coated vesicles (CCVs) that are pinched off and deliver their cargoes into the cell (Lampe et al., 2016; Kaksonen and Roux, 2018).

How the clathrin coat deforms the membrane from a flat patch into a spherical vesicle has long been a matter of debate. Currently, two distinct models have been proposed to explain this process. In the first, the constant curvature model, the clathrin coat directly polymerizes into curved lattices driving invagination, while in the second model, clathrin initially assembles as a flat lattice and is continuously rearranged into a spherical bud while keeping its area constant. In vitro data (Kirchhausen and Harrison, 1981; Dannhauser and Ungewickell, 2012; Kirchhausen et al., 2014) largely support the first model, while cellular data favor the second (Heuser, 2000; Avinoam et al., 2015). Most recently, however, it was shown that both modes of curvature can happen in the same cell, suggesting that cooperating and competing forces will define which model of invagination is followed (Scott et al., 2018). Such forces will include the local protein and lipid content of the CCP and how they contribute to the energetic costs of membrane deformation (Stachowiak et al., 2013; Sorokin et al., 2018).

Flat clathrin lattices are hexagonal arrays, and to gain curvature, rearrangement is needed to convert hexagons into pentagons (Kanaseki and Kadota, 1969), and this in turn requires clathrin exchange (den Otter and Briels, 2011). FRAP experiments have shown that there is extensive exchange between membrane-associated and cytoplasmic clathrin (Wu et al., 2001). This is mediated by cyclin G–associated kinase (GAK; Lee et al., 2006), which is the ubiquitously expressed form of the neuronal protein, auxilin, both of which also act as cofactors for clathrin disassembly during CCV uncoating (Ungewickell et al., 1995; Eisenberg and Greene, 2007). Consistent with this, these proteins have been implicated functionally in early stages of CCP formation (Newmyer et al., 2003).

The energy of clathrin polymerization contributes to membrane deformation (Dannhauser and Ungewickell, 2012; Saleem et al., 2015), and in vitro research showed that CLCs contribute to the tensile strength of the lattice and are required for the deformation of artificial membranes with high bending rigidity (Dannhauser et al., 2015). This impact of the CLCs on the mechanical properties of the clathrin lattice is also reflected in their requirement for CME of large particles such as bacteria (Bonazzi et al., 2011) as well as at sites of increased membrane tension (Boulant et al., 2011). Our recent research revealed that CLCs and specific phosphorylation sites are required for the ligand-stimulated uptake of a subset of G protein–coupled receptors (GPCRs; Ferreira et al., 2012), which contrasted with their dispensable role for uptake of single-pass transmembrane proteins such as transferrin receptor (TfR) and epidermal growth factor receptor in some (Hinrichsen et al., 2003; Huang et al., 2004) but not all cases (Chen et al., 2017). The requirement for CLCs for the uptake of some GPCRs was subsequently verified in vivo (Wu et al., 2016). Importantly, CME of ligand-stimulated GPCRs differs in certain respects from that of constitutive uptake of cargoes such as TfR (Irannejad and von Zastrow, 2014). However, the mechanism or mechanisms by which CLCs contribute to cargo-selective uptake have remained elusive thus far.

In this study, we provide evidence that clustering of cargoes such as the multimembrane-spanning GPCR P2Y_12_ can influence the mode of curvature generation by the clathrin lattice. For CCPs with high amounts of P2Y_12_, this process is dependent on the transition of flat clathrin lattices into spherical CCVs though clathrin rearrangement regulated by phosphorylation of CLCb and is mediated though auxilin in neuronal cells. However, this process is not required for the uptake of TfR, leading us to propose that CCPs invaginate using variable modes of curvature depending on the composition of the cargo that they incorporate.

## Results

### CLCb phosphorylation controls cargo uptake from CCPs

**Figure 1.**
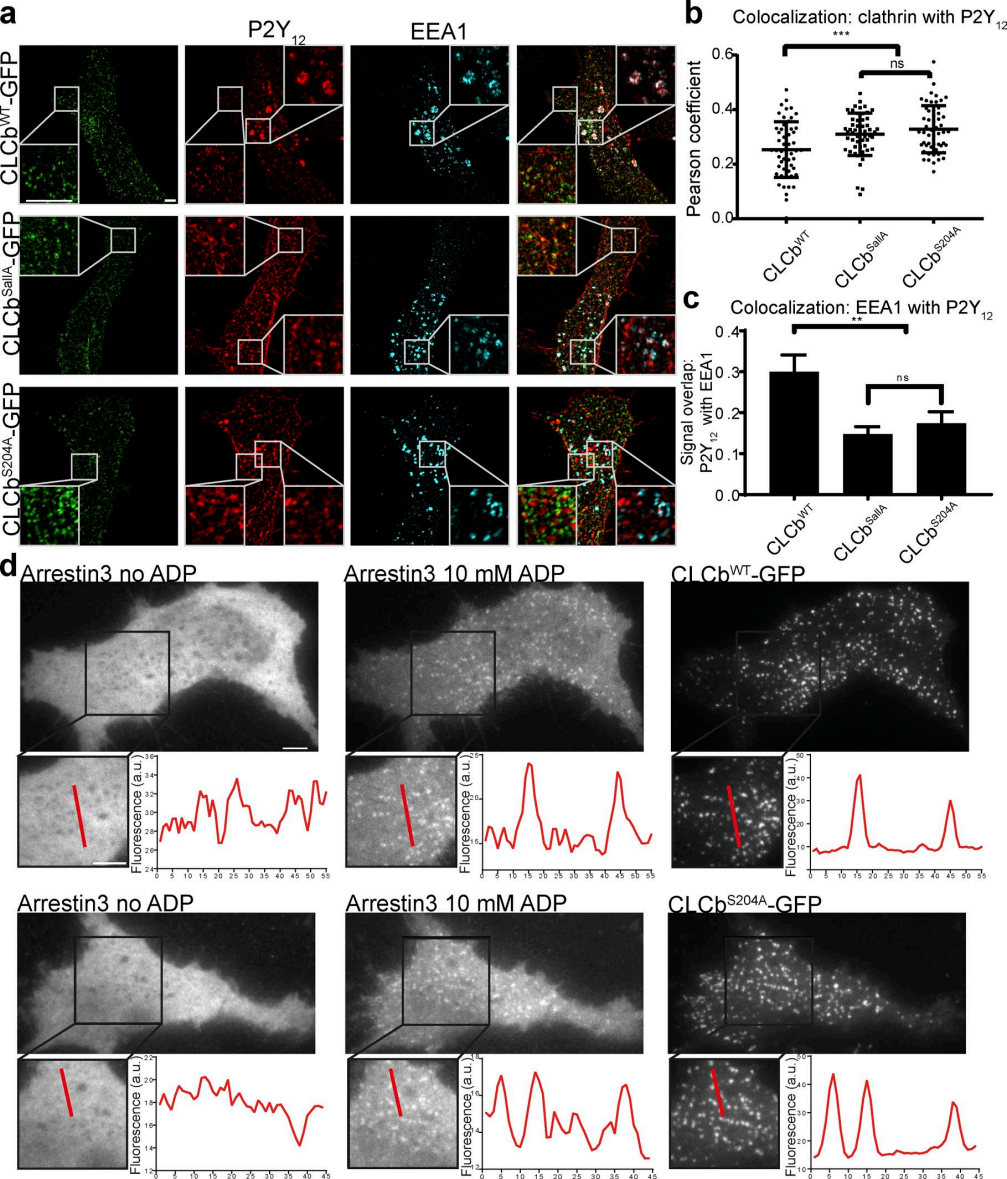
**CLCs control P2Y_12_ uptake. (a)** 1321N1 cells stably expressing HA-tagged P2Y_12_ receptor were transfected with CLCb^WT^-GFP, CLCb^SallA^-GFP, or CLCb^S204A^-GFP, and following incubation in serum-free medium for 1 h and treatment with 10 mM ADP for 10 min, stained with antibodies against the HA tag and EEA1. White boxes show the indicated area at higher magnification. Bars, 5 µm. **(b)** Colocalization measured by Pearson’s coefficient of P2Y_12_ with CLCb^WT^-GFP, CLCb^SallA^-GFP, and CLCb^S204A^-GFP at the plasma membrane. *n* = 50–55 from 24–27 cells. Error bars are mean ± SD. ***, P < 0.001. **(c)** Percent signal overlap between P2Y_12_ with EEA1 from whole Z stacks using image segmentation. *n* = 24–27 cells. Error bars are mean ± SEM. **, P < 0.01. **(d)** 1321N1 cells were transfected with arrestin3-mApple together with CLCb^WT^-GFP or CLCb^S204A^-GFP and imaged with TIRF microscopy before and after treatment with 10 mM ADP; fluorescence was analyzed using line scans. Bars, 5 µm.

Our previous research (Ferreira et al., 2012) using an ELISA-based assay showed that expression of a nonphosphorylatable mutant CLCb where all 19 serines had been mutated to alanines, CLCb^SallA^, inhibited internalization of both the GPCR purinergic receptors P2Y_1_ and P2Y_12_ in 1321N1 astrocytoma cell lines, which stably expressed HA-tagged receptors. By contrast, expression of a mutant with a single point mutation converting serine_204_ to alanine, CLCb^S204A^, inhibited P2Y_12_ uptake but did not affect P2Y_1_ uptake (Ferreira et al., 2012). This indicated that although CLCb phosphorylation is important for the uptake of P2Y_1_, the relevant site is not serine_204_ but some other site or sites. HeLa cells are a well-established model for exploring general mechanisms of endocytosis applicable to multiple cargoes, while 1321N1 cells, because of their neuronal origin, are likely to have specialized machinery required for P2Y_12_ uptake. For this reason, in the experiments described below, we compared CLCb^SallA^ with CLCb^WT^ in HeLa cells when we tested effects on endocytosis that were likely to affect a range of cargoes, and CLCb^S204A^ with CLCb^WT^ when we were specifically measuring P2Y_12_ uptake in 1321N1 astrocytoma cells. It is of note that we see similar results with CLCb^SallA^ and CLCb^S204A^ when we measure uptake of P2Y_12,_ further indicating that phosphorylation of serine_204_ is the key residue in phosphorylation-mediated regulation of uptake of this receptor (Fig. 1; Ferreira et al., 2012).

To more precisely define the requirement for CLCb phosphorylation in P2Y_12_ uptake, we expressed CLCb WT and phosphorylation-deficient mutants in 1321N1 astrocytoma cells that stably express HA-tagged P2Y_12_. Following incubation in serum-free medium and stimulation with 10 mM ADP, the agonist for the P2Y_12_ receptor, cells expressing CLCb^WT^-GFP internalized P2Y_12_ from the plasma membrane into EEA1-positive endosomes (Fig. 1, a–c). In contrast, expression of CLCb^SallA^-GFP led to a decreased colocalization of P2Y_12_ with EEA1 after 10 min ligand stimulation. Consistent with our previous research (Ferreira et al., 2012), expression of CLCb^S204A^-GFP resulted in the same phenotype. Expression of phosphorylation-deficient mutants caused increased colocalization with clathrin at the plasma membrane compared with expression of CLCb^WT^ (Fig. 1, a–c). The increased colocalization of P2Y_12_ with clathrin at the plasma membrane indicates that reduced internalization in the presence of phosphorylation-deficient mutants is likely due to defects in CCP dynamics.

Ligand-stimulated CME of GPCRs differs in certain aspects from that of housekeeping cargoes such as TfR. Arrestins are specialized adaptor proteins that facilitate GPCR uptake, and arrestin3 is specifically required for P2Y_12_ internalization (Kelly et al., 2008). To address whether CLCb phosphorylation affected arrestin3 recruitment, we expressed arrestin3 fused to mApple (mApple-arrestin3) in 1321N1 cells expressing either CLCb^WT^-GFP or CLCb^S204A^-GFP and assessed its ligand-dependent clustering into CCPs. In the absence of ligand, mApple-arrestin3 localized diffusely in the cytoplasm, and it was recruited into CCPs immediately after stimulation regardless of the phosphorylation state of CLCb (Fig. 1 d). Therefore, the receptor is able to enter into CCPs and recruit an essential adaptor protein but is not endocytosed if CLCb cannot be phosphorylated at serine_204_.

**Figure 2.**
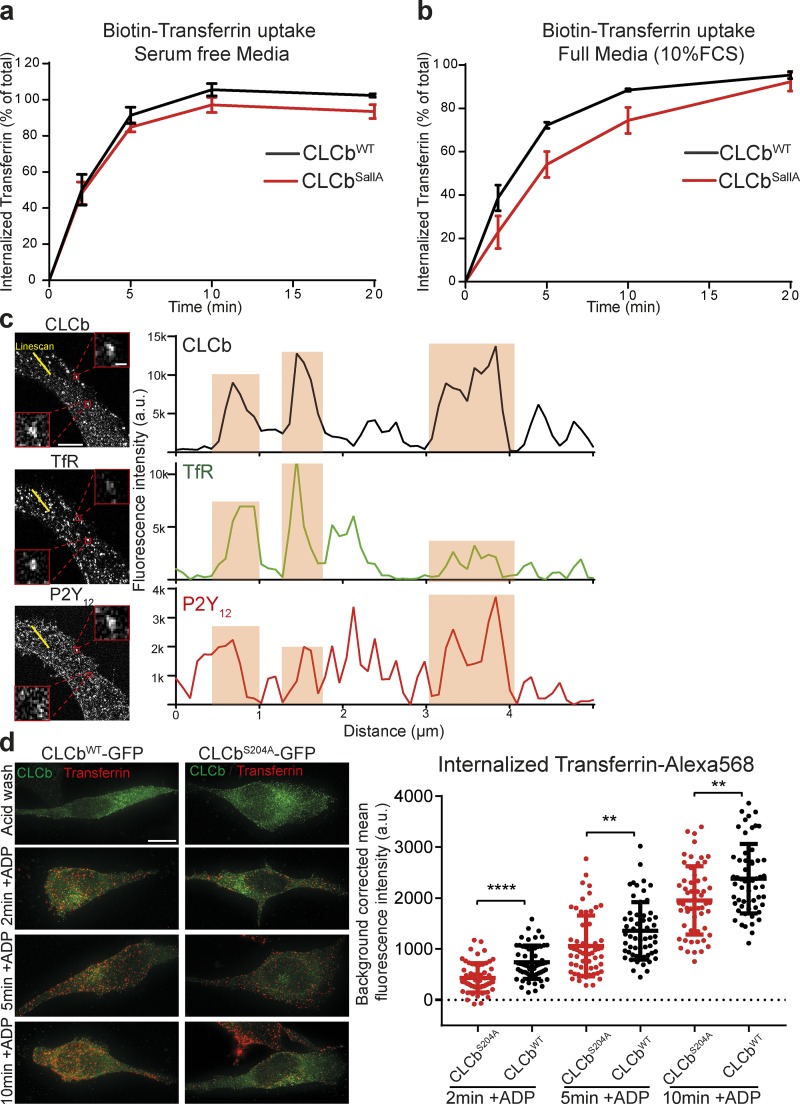
**Transferrin uptake becomes dependent on CLCb phosphorylation in the presence of other cargoes. (a)** HeLa cells expressing CLCb^WT^ and CLCb^SallA^ were serum starved for 1 h, and uptake of biotinylated transferrin in serum-free media was measured using an ELISA assay. Numbers represent mean ± SEM. *n* = 3. **(b)** Uptake of biotinylated transferrin in HeLa cells expressing CLCb^WT^ or CLCb^SallA^ without prior serum starvation and in the presence of full growth media containing 10% FCS. Numbers represent mean ± SEM. *n* = 3. **(c)** 1321N1 cells stably expressing HA-tagged P2Y_12_ receptor were stimulated with ADP for 3 min and stained with antibodies against HA, TfR, and CLCb. Yellow line represents the intensity profiles in the right panels. Boxed areas show examples of CCPs with different amount of cargo packing. Bars: 5 µm (overview); 400 nm (boxed areas). **(d)** 1321N1 cells stably expressing the P2Y_12_ receptor were transfected with either CLCb^WT^-GFP or CLCb^S204A^-GFP, and uptake of fluorescently labeled transferrin in the presence of ADP was determined by fluorescence microscopy. Error bars are mean ± SD. *n* = 75–80 cells for each time point pooled from three independent repeats. **, P < 0.01; ****, P < 0.0001. Bar, 10 µm.

Previous studies (Hinrichsen et al., 2003; Huang et al., 2004) have established that CLCs are dispensable for TfR uptake in contrast with their essential role for GPCR uptake (Ferreira et al., 2012; Wu et al., 2016). In our previous work, expression of phosphorylation-deficient mutants resulted in a mild effect on transferrin internalization when measured at 31°C (Ferreira et al., 2012). However, when measured at 37°C, expression of phosphorylation-deficient mutants in HeLa cells did not affect internalization of biotinylated transferrin compared with cells expressing CLCb^WT^-GFP (Fig. 2 a). In this experiment, we first serum starved the cells, and then we measured internalization of biotinylated transferrin in serum-free medium. This is usual for such assays (Smythe et al., 1992, 1994) because it allows the uptake of a single cargo to be measured in the absence of other cargo molecules. However, cells grown in the presence of serum are exposed to a range of molecules, including GPCR agonists and growth factors, that could stimulate uptake of their cognate cell surface receptors. We were curious as to whether the presence of such cargo molecules might affect TfR uptake in HeLa cells expressing CLCb^SallA^-GFP compared with those expressing CLCb^WT^ -GFP. To test this, we measured uptake of biotinylated transferrin in the presence of media containing 10% FCS. Under these conditions, we observed a decrease in the rate of biotinylated transferrin uptake in cells expressing phosphorylation-deficient CLCb (Fig. 2 b). This indicated that in the presence of other cargoes, efficient TfR internalization becomes dependent on CLCb phosphorylation.

This raised the question as to whether TfR and P2Y_12_ are copackaged in the same CCPs. When we analyzed the colocalization of the P2Y_12_ receptor with TfR in CCPs, after short ligand stimulation with ADP, we found that they partially colocalize into the same clathrin-coated structures (CCSs), albeit to different extents (Figs. 2 c and S1 a; ∼22% of TfR was present in structures positive for P2Y_12_). This suggests that TfR is stochastically packed into most, if not all, CCPs but also that it may be outcompeted by high levels of P2Y_12_. Therefore, stimulation of P2Y_12_ uptake should make TfR uptake sensitive to CLCb phosphorylation at serine_204_ (the site specific for P2Y_12_ uptake). To test this, we measured the uptake of transferrin–Alexa Fluor 568 following costimulation of 1321N1 cells with ADP to stimulate P2Y_12_ uptake. We found that there was a decrease in transferrin–Alexa Fluor 568 uptake in 1321N1 cells expressing CLCb^S204A^-GFP compared with those expressing CLCb^WT^-GFP (Fig. 2 c). Together, these data indicate that TfR and P2Y_12_ receptors can be copackaged in the same CCPs following stimulation with ADP, and when this happens, uptake of these endocytic structures becomes dependent on CLCb phosphorylation.

### CLCb phosphorylation and cargo influences lifetime dynamics of CCPs

Total internal reflection fluorescence (TIRF) microscopy is a well-established technique to measure the dynamics of CCP initiation and growth. The lifetime of CCPs is indicative of their functionality, and it has been shown that bona fide CCPs that recruit cargo, grow, invaginate, and pinch off to form CCVs have a broad lifetime distribution (20–60 s). By contrast, short-lived CCPs with a lifetime of 10–20 s are predominately abortive events that disassemble rapidly before they can capture cargo, gain curvature, or recruit dynamin (Merrifield et al., 2002; Ehrlich et al., 2004; Loerke et al., 2011; Taylor et al., 2012; Aguet et al., 2013). We compared the lifetime dynamics of CCPs in the absence and presence of arrestin3 (as a measure of P2Y_12_ recruitment to CCPs) and transferrin. As observed previously (Aguet et al., 2013), the incorporation of transferrin stabilized nascent CCPs as evidenced by their broad lifetime distribution, while CCPs that failed to take up this housekeeping cargo displayed a predominately short lifetime of <20 s (Fig. S1 b). Stimulation with ADP resulted in recruitment of arrestin3 to ∼50% of CCPs, which showed a similar broad lifetime distribution (Fig. S1 c). It is noteworthy that the productive CCP population containing arrestin3 was almost twice as long lived as the population containing transferrin (63.09 s versus 31.52 s). Therefore, CCPs that mainly internalize TfR have a higher turnover rate and could compensate for the decreased uptake of TfR that is copackaged with the P2Y_12_ receptor and is slower to internalize. It was recently shown that CCPs with longer lifetimes tend to gain curvature using the constant area mode, while CCPs with shorter lifetimes tend more toward the constant curvature pathway (Scott et al., 2018). Therefore, the difference in the lifetime dynamics of CCPs trafficking transferrin or arrestin3 suggests different modes of CCP assembly.

Previous studies have implicated the CLCs in the stability of the clathrin lattice (Schmid et al., 1982; Ungewickell, 1983; Ybe et al., 1998, 2007; Wilbur et al., 2010), and their depletion was shown to affect the lifetime dynamics of CCPs (Mettlen et al., 2009; Boulant et al., 2011). We confirmed that CLCs affect lattice stability in 1321N1 cells by depleting both CLCa and CLCb using siRNA and analyzing the lifetime dynamics of CCPs after addition of fluorescently labeled transferrin. Depletion of both CLCs caused an increase in those short-lived events that fail to incorporate transferrin (Fig. S2 a). There was also an increased number of short-lived CCPs in HeLa cells expressing CLCb^SallA^ or 1321N1 cells expressing CLCb^S204A^ under ligand-stimulated conditions compared with cells expressing CLCb^WT^-GFP (Fig. S2, b and c). Consistent with previous results (Loerke et al., 2009; Aguet et al., 2013), these short-lived events failed to recruit dynamin, cargo, and adaptor proteins including eps15, epsin2, and CALM, all of which have been shown to have important roles in CCP maturation (Figs. S2 and S3; Chen et al., 1998; Benmerah et al., 1999; Tebar et al., 1999; Meyerholz et al., 2005; Boucrot et al., 2012; Sahlender et al., 2013; Messa et al., 2014; Holkar et al., 2015; Miller et al., 2015; Ma et al., 2016). Additionally, the short-lived events also failed to recruit arrestin3 when P2Y_12_ uptake was stimulated in 1321N1 cells (Fig. S2 c). Strikingly, when we investigated the recruitment of the ubiquitously expressed GAK, we found that it was recruited to short-lived events (Fig. S2 d). GAK regulates clathrin lattice rearrangement as well as uncoating, and its presence at these short-lived events could indicate that they are either abortive CCPs that fail to mature and get actively disassembled or that they are CCVs that have recruited GAK but fail to uncoat and transiently visit the TIRF field. Together, these data show that phosphorylation of CLCb is required for the maturation of a population of CCPs when cells are stimulated with ligand and that inability to phosphorylate CLCb increases the number of short-lived clathrin structures that recruit GAK.

### CLCb phosphorylation controls invagination

**Figure 3.**
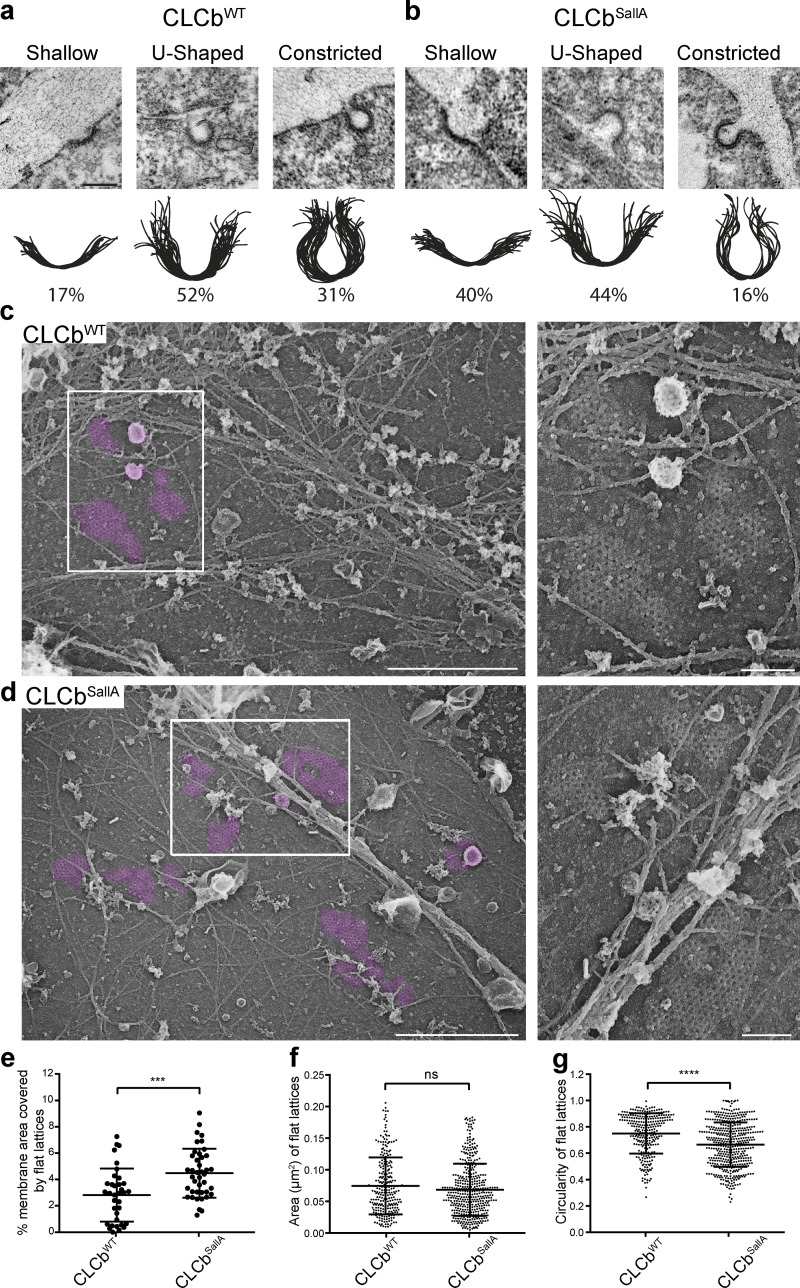
**CLCb phosphorylation regulates CCP invagination. (a and b)** HeLa cells expressing either CLCb^WT^-GFP (a) or CLCb^SallA^-GFP (b) were prepared for standard resin-embedded thin-section transmission EM, and individual CCPs were traced by hand using Illustrator and binned into shallow, U-shaped, or constricted. Bar, 200 nm. *n* = 67–88. **(c and d)** Survey view of the cytoplasmic surface of the plasma membrane in unroofed HeLa cells expressing either CLCb^WT^-GFP or CLCb^SallA^-GFP. Clathrin lattices are pseudocolored in purple. Insets corresponding with boxed regions in c and d show high-magnification views. Bars: 1 µm (main images); 200 nm (insets). **(e)** Percent membrane area covered by flat lattices. *n* = 35–41. ***, P < 0.001. **(f)** Area of all individual flat lattices from 35–41 different membranes. *n* = 278–461. **(g)** Circularity of all individual flat lattices as defined by Circularity = $\;4\pi \times \left(\frac{Area}{Perimeter^{2}}\right)$, with a value of 1.0 indicating a perfect circle. *n* = 278–461. ****, P < 0.0001. Values represent mean ± SD.

To gain a better understanding of how CLCb phosphorylation controls maturation of a proportion of CCPs, we wanted to investigate whether there was a correlation between CLCb phosphorylation and CCP invagination. HeLa cells expressing CLCb^WT^ and CLCb^SallA^ were grown in media containing 10% FCS, processed for thin-section EM, and the morphology of CCPs was assessed. As expected, CCPs from cells expressing CLCb^WT^ showed a broad distribution of morphologies ranging from shallow to U shaped and constricted (Fig. 3 a). Conversely, CCPs in cells expressing CLCb^SallA^ showed a shift in distribution toward shallow CCPs compared with CLCb^WT^ (Fig. 3 b). Failure to phosphorylate CLCb is thus reflected in an increased population of CCPs that fail to invaginate in the presence of FCS, where uptake of a range of cargoes is stimulated, some of which are dependent on CLCb phosphorylation.

Thin-section EM is a powerful tool to investigate CCP morphologies, but it is unable to detect flat clathrin lattices. The latter are larger and longer lived than classical CCPs and have been shown to control the uptake of lysophosphatidic acid (LPA; Leyton-Puig et al., 2017) and CCR5 receptors (Grove et al., 2014). It is noteworthy that LPA is a major constituent of serum (Eichholtz et al., 1993) and might contribute to the effect of serum on TfR uptake in the presence of phosphorylation-deficient CLCb. To investigate flat clathrin lattices, we employed metal replica EM of the plasma membrane from unroofed HeLa cells grown in the presence of FCS and expressing either CLCb^WT^ or CLCb^SallA^. We found that flat lattices as well as deeply invaginated CCPs were present in both CLCb^WT^- and CLCb^SallA^-expressing cells (Fig. 3, c and d). In cells expressing CLCb^SallA^, there was an increase in the percentage of the plasma membrane that is covered by flat lattices (Fig. 3 e). Interestingly, although there was no significant difference in the mean area of flat lattices in either CLCb^WT^- or CLCb^SallA^-expressing cells (Fig. 3 f), the lattices formed in the presence of the phosphorylation-deficient CLCb mutant had a more irregular shape, which is apparent in a significant decrease in their circularity (Fig. 3 g). The increase in shallow CCPs is therefore also reflected in an increase in flat clathrin lattices of more irregular shapes. These flat lattices are unlikely to represent the short-lived events observed by TIRF microscopy but rather point to defects in lattice rearrangement and curvature generation likely due to altered interactions of GAK with phosphorylation-deficient CLCb.

### CLCb phosphorylation is required for rapid clathrin exchange

**Figure 4.**
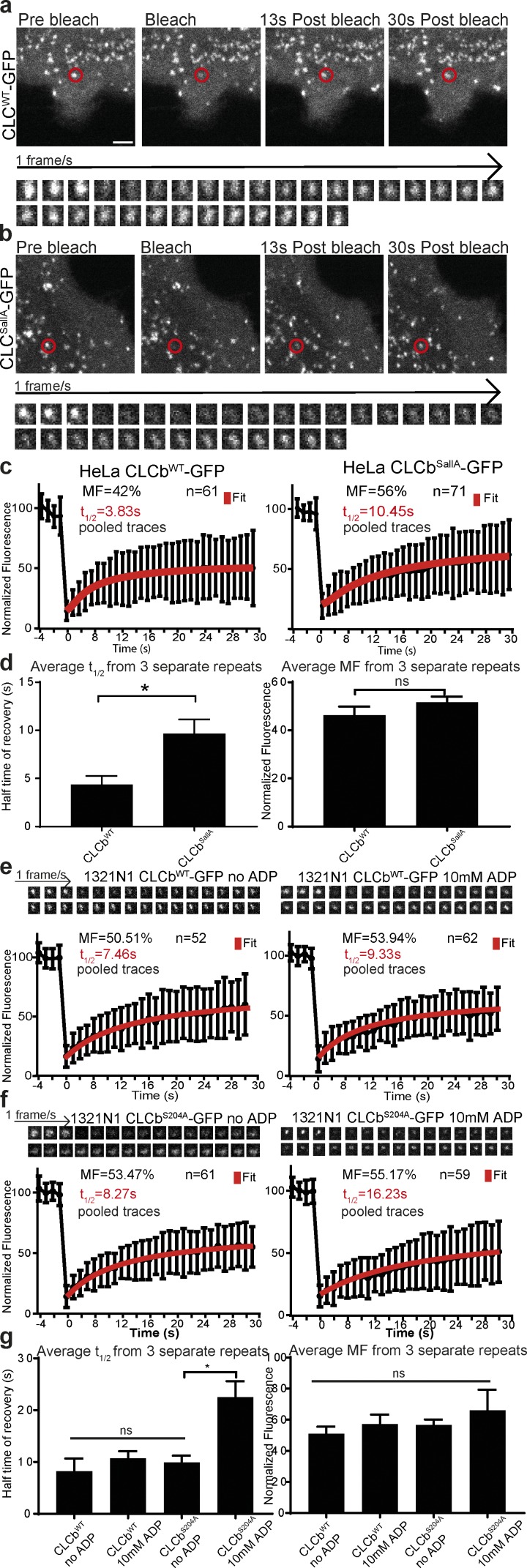
**CLCb phosphorylation regulates clathrin lattice rearrangements. (a and b)** Fluorescence from individual CCSs at the plasma membrane of HeLa cells grown in the presence of 10% FCS and expressing either CLCb^WT^-GFP (a) or CLCb^SallA^-GFP (b) was photobleached, and recovery of the signal was measured for 30 s at one frame per second. Bar, 2 µm. **(c)** Normalized recovery traces were fitted to cumulative data from three repeats with a hyperbola function of *y*(*t*) = *offset* + *MF* * *t*/(*t* + *t*_1/2_ ) with Offset = bleaching efficiency and *t*_1/2_ = halftime of recovery. Error bars are mean ± SD. **(d)** Average values from each of the three separate repeats from c. *n* = 3 with 16–23 traces from each repeat. Error bars are mean ± SEM. **(e and f)** 1321N1 cells were transfected with either CLCb^WT^-GFP or CLCb^S204A^-GFP, and fluorescence recovery of a single CCS was measured following incubation in serum-free medium before and directly after addition of 10 mM ADP. Top: Typical traces with the cumulative data from three independent repeats. Error bars are mean ± SD. **(g)** Average values from each of the repeats shown in e and f. Error bars show mean ± SEM. *n* = 3 with 16–26 traces from each repeat. *, P < 0.05.

To convert a flat lattice to a more invaginated CCP, clathrin needs to be rearranged to allow the incorporation of pentagons, which increases curvature (Kanaseki and Kadota, 1969; den Otter and Briels, 2011). To address whether the decrease in receptor uptake and curvature generation might be due to a defect in clathrin rearrangement, FRAP experiments were performed on individual CCSs at the plasma membrane of HeLa cells expressing either CLCb^WT^-GFP or CLCb^SallA^-GFP in the presence of 10% FCS (Fig. 4, a and b). In HeLa cells expressing CLCb^WT^-GFP, the fluorescence signal recovered rapidly with a *t*_1/2_ of 3.83 s, in agreement with previous research (Avinoam et al., 2015). However, this rapid recovery was greatly reduced in HeLa cells expressing CLCb^SallA^-GFP as evident by the increased *t*_1/2_ of 10.45 s with no significant effect on the mobile fraction (MF; Fig. 4, c and d), indicating a defect in clathrin exchange and lattice rearrangement.

We have previously shown that phosphorylation of serine_204_, mediated by GPCR kinase 2 (GRK2), is a specific requirement for the uptake of the P2Y_12_ receptor (Ferreira et al., 2012). We therefore wanted to address whether defects in clathrin rearrangement in the presence of CLCb^S204A^ could be responsible for the reduction in P2Y_12_ uptake. In the absence of ligand, the P2Y_12_ receptor is retained at the plasma membrane and is only endocytosed upon ADP stimulation. We therefore treated 1321N1 cells expressing the P2Y_12_ receptor with ADP and performed FRAP experiments before and directly after ligand stimulation. In 1321N1 cells expressing CLCb^WT^-GFP, ADP stimulation only mildly affected the recovery half-time, with a change in the *t*_1/2_ from 7.46 s to 9.33 s and no apparent change in the MF (Fig. 4, e and g). In the absence of ADP, the *t*_1/2_ in cells expressing CLC^S204A^-GFP was similar to that of cells expressing CLC^WT^-GFP, with a *t*_1/2_ of 8.27 s (Fig. 4, f and g). The difference in the half-time of recovery in HeLa cells expressing CLCb^WT^-GFP (3.83 s) and 1321N1 cells expressing CLCb^WT^-GFP (7.46 s) is likely due to differences in the expression of endocytic factors such as auxilin and GAK, which have both been implicated in clathrin exchange (Wu et al., 2001; Yim et al., 2005; Lee et al., 2006). Strikingly, however, upon ligand stimulation in cells expressing CLCb^S204A^-GFP, the *t*_1/2_ increased significantly to 16.23 s, while the MF remained unaffected (Fig. 4, f and g). The effect on clathrin rearrangement is thus ligand dependent in cells expressing CLCb^S204A^-GFP and is only manifest once uptake of the P2Y_12_ receptor is stimulated.

### Auxilin is required for P2Y_12_ uptake

**Figure 5.**
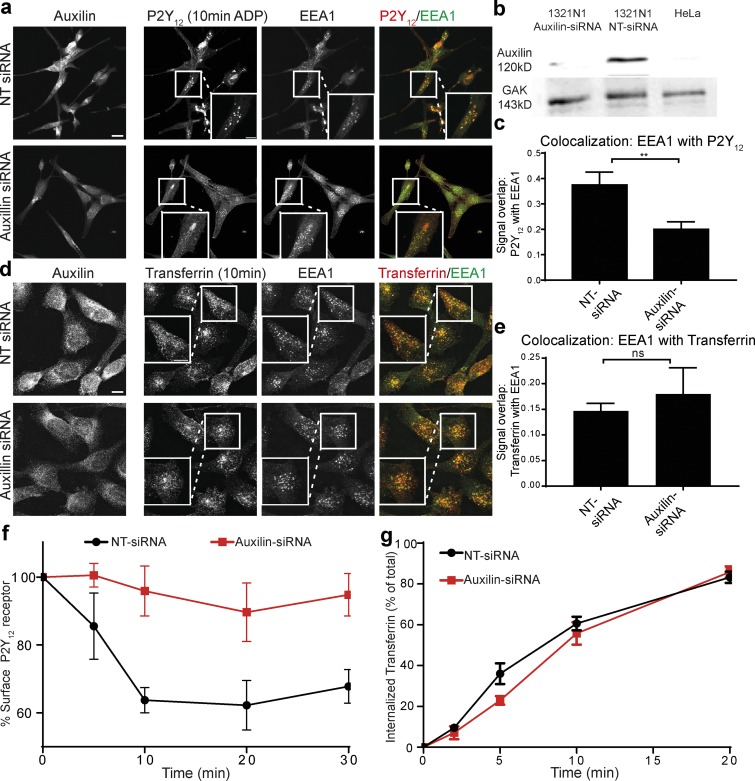
**Auxilin is required for efficient P2Y_12_ uptake. (a)** 1321N1 cells were treated with control NT siRNA or siRNA targeting auxilin, treated with 10 mM ADP for 10 min to stimulate P2Y_12_ uptake, and stained with antibodies against auxilin, P2Y_12_, and EEA1. White boxes show an enlarged area. **(b)** Western blots showing levels of auxilin and GAK in HeLa cells and in 1321N1 cells following treatment with NT siRNA and siRNA targeting auxilin. Equal amounts of protein were loaded in each lane. **(c)** Quantification of the percent signal overlap between P2Y_12_ with EEA1 from whole Z stacks using the image segmentation tool SQUASSH. **, P < 0.01. **(d)** 1321N1 cells were treated with control NT siRNA or siRNA targeting auxilin, treated with Alexa Fluor 568 transferrin (5 µg/ml) for 10 min, and stained with antibodies against auxilin and EEA1. White boxes show an enlarged area. Bars, 10 µm. **(e)** Quantification of the percent signal overlap between Alexa Fluor 568 transferrin with EEA1 from whole Z stacks using the image segmentation tool SQUASSH. **(f)** ADP-induced (10 mM) loss of surface P2Y_12_ receptor was measured by ELISA in 1321N1 cells treated with NT siRNA or auxilin siRNA. *n* = 3. **(g)** Uptake of biotinylated transferrin was measured by ELISA in 1321N1 cells treated with NT siRNA or auxilin siRNA. Numbers represent mean ± SEM. *n* = 3.

Since auxilin and GAK are required for efficient clathrin rearrangement and since auxilin expression is high in neuronal cells (Fig. 5 b), a prediction of these results is that auxilin and/or GAK should also be required for P2Y_12_ uptake in 1321N1 astrocytoma cells. To test this, we depleted 1321N1 cells of auxilin using siRNA (Fig. 5 b), and P2Y_12_ uptake was stimulated by addition of ADP for 10 min. In cells treated with nontargeting (NT) control siRNA, the receptor was readily taken up into EEA1-positive endosomes, while in cells depleted of auxilin, the P2Y_12_ receptor was mainly localized at the plasma membrane (Fig. 5, a and c), similar to cells expressing either CLCb^SallA^-GFP or CLCb^S204A^-GFP (Fig. 1 c). To verify these results biochemically, we used an ELISA assay to determine the level of receptor uptake after ADP stimulation. In agreement with the microscopy data, cells treated with NT siRNA readily internalized the P2Y_12_ receptor from the plasma membrane, while receptor uptake was almost completely abolished in cells depleted of auxilin (Fig. 5 f). By contrast, depletion of auxilin had only a very mild effect (evident in an ELISA assay after 5 min only) on transferrin uptake when measured under serum-free conditions (Fig. 5, d, e, and g). This indicates that auxilin-mediated clathrin rearrangement is a requirement for the uptake of the P2Y_12_ receptor but not for the classical CME cargo transferrin in this cell line. Interestingly, in 1321N1 cells, GAK is expressed at levels comparable with those found in HeLa cells, where auxilin is expressed at almost undetectable levels (Fig. 5 b). However, GAK does not appear to be capable of compensating for loss of auxilin in the astrocytoma cells. This is consistent with in vivo research, which supports independent and overlapping functions of GAK and auxilin (Yim et al., 2010).

## Discussion

### Cargo regulates CCP invagination

The aim of our study was to understand the underlying mechanism for the differential requirement for CLCs and their phosphorylation in the uptake of the GPCR P2Y_12_ versus TfR. It has been well established that CLCs are not generally required for the uptake of transferrin (Hinrichsen et al., 2003; Huang et al., 2004), which is often used as a canonical measure of CME because the route followed by receptor and ligand has been so well defined (Hopkins and Trowbridge, 1983; Watts, 1985). However, we show that uptake of transferrin is sensitive to the presence of other cargoes when they are copackaged into the same endocytic structures. Stimulation of 1321N1 cells with ADP results in the clustering of P2Y_12_ in CCPs, and we have shown that TfR can be copackaged with P2Y_12_. In line with our previous research (Ferreira et al., 2012), we show that uptake of P2Y_12_ is regulated by CLCb phosphorylation at serine_204_. When P2Y_12_ uptake is stimulated, transferrin uptake is also decreased in cells expressing CLCb^S204A^. A similar result is observed in HeLa cells expressing CLCb^SallA^, where all 19 serine residues are mutated to alanine. Cells expressing this phosphorylation-deficient CLCb mutant show a delay in transferrin uptake in the presence of FCS, which contains ligands for other transmembrane receptors. While the phosphorylation sites required for uptake of other cargoes are not yet defined, our data show that transferrin uptake can become more dependent on phosphorylation of CLCb when the local cargo composition of CCPs is changed (e.g., by clustering of GPCRs that are dependent on CLCs).

**Figure 6.**
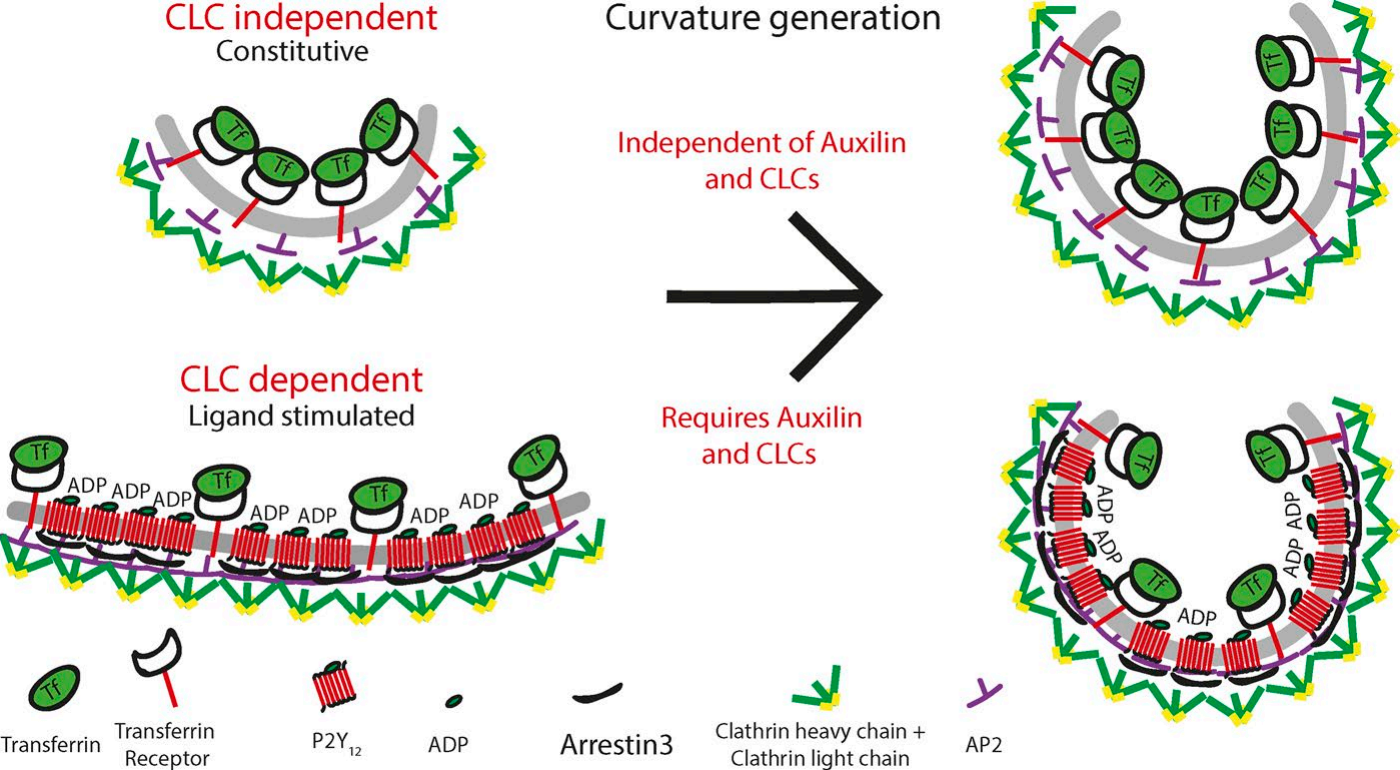
**Model for cargo regulation of CCP assembly mode regulated by CLC phosphorylation and auxilin.** CME of single-pass transmembrane proteins such as TfR that are constitutively internalized is independent of CLCs and lattice rearrangement when measured in the absence of other cargo. Following ligand stimulation, cargo such as P2Y_12_ will recruit specialized adaptor proteins and oligomerize, packing tightly into CCPs, which will result in some exclusion of other cargo. Maturation of these CCPs then becomes dependent on CLCs and their ability to be phosphorylated as well as auxilin-mediated clathrin exchange, likely by contributing to the flat-to-curved transition of CCPs containing high amounts of P2Y_12_.

The sorting of cargo proteins into distinct CCPs has been a matter of debate (Keyel et al., 2006). In this study, we show that TfR and P2Y_12_ partially colocalize into the same CCPs, albeit to different extents. This is consistent with previous studies that showed that receptors are stochastically recruited into CCPs (Keyel et al., 2006; Lampe et al., 2014). However, high levels of P2Y_12_ are likely to outcompete TfR from CCPs due to their oligomeric nature (Fig. 6). Under ligand-stimulated conditions, ∼50% of productive CCPs recruited arrestin3 with a lifetime of ∼60 s, while CCPs trafficking TfR had a mean lifetime of ∼30 s. Therefore, it is possible that TfR uptake “catches up” through CCPs that do not recruit P2Y_12_ or arrestin3 and have a higher turnover rate. This may explain why TfR uptake is reduced and not abolished in cells expressing phosphorylation-deficient CLCb mutants.

Our study has revealed that CLC phosphorylation plays an essential role in CCP invagination, leading to efficient internalization of CCS containing high amounts of P2Y_12_. The two models of membrane curvature generation during CCP invagination, constant area versus constant curvature, are conceptually quite different, requiring different mechanisms of lattice assembly. Both behaviors have however been observed in CCPs in SK-MEL-2 cells (Scott et al., 2018). Furthermore, a recent study argues for the assembly of flat lattices to ∼70% of their final size before curvature is initiated (Bucher et al., 2018). Curvature generation during CME is likely to be a complex process with different modes of membrane bending occurring in parallel, reflecting the energetics of coat assembly relative to competing forces. It has been shown in vitro that high membrane tension as well as rigidity directly opposes polymerization of clathrin into curved vesicles (Saleem et al., 2015). However, increased membrane tension in cellular systems is overcome by the actin cytoskeleton and requires CLCs to allow CME to occur (Boulant et al., 2011). It is reasonable to propose that in cases where the polymerization energy of the clathrin triskelia is not sufficient to deform the membrane directly, it will initially assemble as a flat lattice, whereas membranes that are easier to deform might follow the path of constant curvature, directly polymerizing into spherical vesicles. This is consistent with results from a recent study showing that increased tension of the plasma membrane inhibits its deformation and leads to an increase in flat clathrin lattices that fail to generate curvature (Bucher et al., 2018).

The two main factors that oppose local membrane deformation are its lateral tension as well as its rigidity. Membrane rigidity will depend on the local lipid and protein composition, and thus, cargo composition inside a CCP will have a significant impact on the biophysical properties of the membrane (Stachowiak et al., 2013). It is therefore possible that ligand-stimulated clustering of multimembrane-spanning GPCRs alters the local properties of the plasma membrane and biases the mechanism of invagination of CCPs toward the constant area mode. Rearrangement of the lattice is then driven by auxilin (in neuronal cells) and is dependent on the capacity of CLCb to be phosphorylated. CLCs could be needed to increase lattice rigidity, similar to the role of Sec13 in the incorporation of GPI-linked cargoes, predicted to drive opposing curvature, into COPII vesicles on the secretory pathway (Copic et al., 2012). An alternative, although not mutually exclusive, role for CLCb phosphorylation would be to efficiently recruit GAK for lattice rearrangement (see below).

It is also possible that recruitment of specialized endocytic effector proteins influences which mode of invagination is followed. Ligand-stimulated uptake of GPCRs requires the recruitment of the adaptor family of arrestins as well as ubiquitination and interactions of PDZ domains with the cytoskeleton (Hanyaloglu and von Zastrow, 2008). Notably, ligand-stimulated endocytosis of GPCRs results in altered CCP dynamics, with an increased lifetime of CCPs positive for arrestin3 compared with those that are positive for TfR as demonstrated in this study and in previous ones (Henry et al., 2012; Soohoo and Puthenveedu, 2013). Strikingly, a recent study has shown that even though both modes of curvature coexist in SK-MEL-2 cells, CCPs with longer lifetimes tend to gain curvature using the constant area mode, while CCPs with shorter lifetimes tend more toward the constant-curvature pathway (Scott et al., 2018). This further supports the notion that cargo proteins influence the mode of curvature generation of CCPs.

### Phosphorylation of CLCb alters interaction with GAK and lattice rearrangement

How does CLCb phosphorylation control cargo uptake? The recruitment of cargo into CCPs as well as the recruitment of a wide range of adaptor proteins is unaffected by CLCb phosphorylation. Therefore, the defect in receptor uptake is likely to occur during CCP maturation. Lifetime analysis of CCPs revealed an increase in short-lived events with a lifetime of 10–20 s in the background of phosphorylation-deficient CLCb. The nature of these events is somewhat unclear, and they could either be abortive CCPs that fail to mature or they could be CCVs that transiently visit the TIRF field and have not yet uncoated. Importantly, these short-lived events were positive for the clathrin rearranging and uncoating protein GAK but did not recruit the GTPase dynamin2 or a wide array of adaptor proteins (such as eps15, CALM, arrestin3, or epsin2). Their increased appearance in the CLCb mutant could be due to altered interactions with GAK, leading to a delay in uncoating. This is in agreement with previous results showing that CLCb phosphorylation modulates GAK binding to clathrin cages (Ferreira et al., 2012) as well as studies that have implicated CLCs in the uncoating of CCVs (Schmid et al., 1984; Young et al., 2013). However, it is also possible that these short-lived events are abortive events and recruit GAK in its capacity as clathrin chaperone and/or to promote rapid disassembly in the absence of cargo and dynamin. In support of this, depletion of GAK increases the turnover of abortive CCPs (Mettlen et al., 2009). Depletion of both GAK and auxilin in HeLa M cells, where both are expressed, reduces CCP formation at the cell surface by 50% and increases nonproductive cage assembly in the cytoplasm (Hirst et al., 2008), further supporting a role for GAK and auxilin in chaperoning clathrin to the cell surface. Both possibilities point toward an altered interaction of GAK with short-lived CCPs that are increased when CLCb cannot be phosphorylated.

The altered interaction of phosphorylation-deficient CLCb mutants with GAK appears to have a profound effect on clathrin rearrangements during the invagination of maturing pits. This rearrangement is crucial for flat lattices to gain curvature, and in line with this, cells expressing phosphorylation-defective CLCb mutants have an increased number of shallow CCPs as well as an increased number of flat clathrin lattices of more irregular shape. Flat lattices have been shown to be active sites of endocytosis by continuous budding of CCVs from their edges (Lampe et al., 2016) and to be important for the uptake of GPCRs (Grove et al., 2014; Leyton-Puig et al., 2017). For these lattices to invaginate, pentagons have to be incorporated into the flat hexagonal array, and this requires clathrin rearrangement mediated by GAK and auxilin (Lee et al., 2006). Our FRAP data indicate that the half-time of recovery of CLCb^SallA^-GFP is significantly slower than that of CLCb^WT^-GFP. Cells expressing CLCb^S204A^ reveal a similar delay in FRAP but, strikingly, only after stimulation of P2Y_12_ internalization, indicating that CLCs are important for clathrin exchange and that following ligand-induced clustering, lattice rearrangement is required for receptor uptake. Auxilin and GAK are crucial factors for clathrin exchange, and their depletion has been shown to drastically reduce exchange of clathrin at endocytic sites (Greener et al., 2001; Lee et al., 2006). The reduction in P2Y_12_ uptake after auxilin depletion in 1321N1 cells further reinforces the requirement for clathrin exchange for the maturation of these endocytic sites. It also links auxilin-mediated clathrin exchange to early stages of CCP maturation in a cargo-specific manner.

By defining a molecular mechanism by which CLCb phosphorylation and GAK can regulate cargo-specific invagination, our data support a model whereby cargo can determine the mode of CCP assembly. Additionally, the recruitment of adaptor proteins during ligand-stimulated CME could also influence the mode of CCP assembly (Keyel et al., 2006). This idea is supported by a recent study showing that high levels of AP2µ2 lead to the formation of flat lattices (Dambournet et al., 2018). High concentrations of cargo within CCPs would also lead to an increased recruitment of AP2 and bias curvature toward the constant area model compared with CCPs with lower amounts of cargo loading. The assembly of flat lattices followed by lattice rearrangement, regulated by CLCb phosphorylation, would then be required for invagination. This is further supported by the specific effect of auxilin knockdown on P2Y_12_ uptake in 132N1 cells compared with a very mild effect on transferrin and explains why previous studies may not have detected significant effects of auxilin or GAK knockdown on transferrin uptake in other cell types (Zhang et al., 2005; Hirst et al., 2008).

Taken together, these data support a model in which the local concentration and composition of cargo inside a CCP modulates the way it can be deformed (Fig. 6). For cargo such as the P2Y_12_ receptor, CCPs would assemble as flat lattices that require clathrin rearrangement and CLCb phosphorylation. However, for cargo such as transferrin, CCPs could invaginate independently of lattice rearrangement and the CLCs. Importantly, we did not observe separation of cargoes into specialized CCPs, as evidenced by the decrease in transferrin uptake under ligand-stimulated conditions in the background of phosphorylation-deficient CLCb. This suggests that depending on the environment in which the cell finds itself, CCPs will form by variable curvature (Scott et al., 2018), which would be defined, at least in part, by the nature of the cargo incorporated by CCPs.

## Materials and methods

### Cell culture and transfection

HeLa cells and 1321N1 cells were cultured in DMEM supplemented with 10% FCS, glutamine, and penicillin/streptomycin. 1321N1 cells stably expressing the P2Y_12_ receptor with N-terminal HA tag were a gift from S. Mundell and were grown in the presence of G418 as described by Mundell et al. (2006). HeLa cells stably expressing CLCb^WT^-GFP and CLCb^SallA^-GFP were cultured as described previously (Ferreira et al., 2012), and expression of CLCb was induced by addition of 1 mg/ml doxycycline overnight before each experiment. The construct encoding arrestin3-mApple was a gift from M. von Zastrow (University of California, San Francisco, San Francisco, CA). Constructs encoding dynamin2-mCherry, GAK-mCherry, eps15-mCherry, CALM-mCherry, and epsin2-mCherry were gifts from C. Merrifield and acquired from Addgene as described by Taylor et al. (2011). Transient transfection of HeLa cells expressing CLCb, WT, and mutants with constructs encoding dynamin2-mCherry, GAK-mCherry, eps15-mCherry, CALM-mCherry, and epsin2-mCherry was performed using electroporation with the NeonR system the day before imaging. Transfection of 1321N1 cells with constructs encoding CLCb^WT^-GFP, CLCb^SallA^-GFP, CLCb^S204A^-GFP, AP2-GFP, or arrestin3-mApple was performed using Polyfect according to the manufacturer’s guidelines.

For CLC siRNA experiments, 1321N1 cells were split into 6-cm dishes, and the next day, when ∼50% confluent, they were transfected with CLC or NT siRNA (final concentration, 200 nM) using Lipofectamine 2000 and left overnight. The next day, the cells were transfected again with siRNA as before, together with plasmid DNA encoding AP2-GFP. Cells were left for two more days before TIRF imaging. For knockdown of auxilin, 1321N1 cells were grown in 10-cm dishes, and the following day, at ∼30% confluency, cells were transfected with auxilin or NT siRNA at 200 nM final concentration using oligofectamine. Cells were left for 48 h and transfected again with the same siRNAs and used for ELISA assays the following day. Auxilin was detected by immunofluorescence and Western blotting using anti-auxilin antibodies (rabbit; anti-DNAJC6; ab103321; Abcam). EEA1 was detected using mouse anti-EEA1 antibodies (ab70521; Abcam). GAK was detected on Western blots using rabbit antibody from Abcam (ab115179).

The siRNAs had following sequences and were characterized previously: siRNA CLCa, 5′-AAAGACAGUUAUGCAGCUAUU-3′ (Ferreira et al., 2012); siRNA CLCb, 5′-AAGCGCCAGAGUGAACAAGUA-3′ (Ferreira et al., 2012); and siRNA auxilin, 5′-UAUGUUACCUCCAGAAUUA-3′ (Hirst et al., 2008).

### Confocal immunofluorescence and colocalization

1321N1 cells were grown on coverslips and transfected with either CLCb^WT^-GFP, CLCb^SallA^-GFP, or CLCb^S204A^-GFP as described above. On the following day, cells were serum starved for 1 h in the presence of 0.1 U/ml apyrase, stimulated with 10 mM ADP for 10 min, and immediately fixed with 4% PFA. The PFA was quenched using 50 mM NH_4_Cl in PBS, and nonspecific binding was blocked with 1% BSA. Cells were permeabilized using 0.1% Triton X-100. Cells were treated with antibodies against HA (rat; clone 3F10; Roche) and EEA1 (rabbit; clone 1G11; Abcam), and then they were stained using the corresponding secondary antibodies conjugated to Alexa Fluor 561 or Alexa Fluor 647 (Thermo Fisher Scientific). Coverslips were mounted onto microscopy slides and imaged using a Zeiss LSM880 AiryScan confocal microscope with a Plan Apochromat 63× 1.4 NA oil lens. Whole Z stacks were taken at 200-nm intervals.

Colocalization of the HA signal with the CLCb signal was analyzed at the plane of the plasma membrane by choosing three to five random regions (depending on cell size) of 250 × 250 pixels in each channel and determining the Pearson’s correlation coefficient between them using ImageJ (National Institutes of Health). Overlap of the HA signal with EEA1 was determined by image segmentation using the ImageJ plugin SQUASSH (Rizk et al., 2014) on maximum-intensity projections of collapsed Z stacks.

For colocalization of P2Y_12_ and TfR with 1321N1, cells were stimulated with 10 mM ADP for 3 min and stained against the HA (rat; clone 3F10; Roche), TfR (mouse; B3/25; ATCC), and CLCb (rabbit; sc-28277; Santa Cruz Biotechnology, Inc.) with the corresponding secondary antibodies conjugated to Alexa Fluor 488, Alexa Fluor 561, or Alexa Fluor 647. Coverslips were mounted onto microscopy slides and imaged using an LSM880 AiryScan confocal microscope with a Plan Apochromat 63× 1.4 NA oil lens at the plane of the plasma membrane. Colocalization of P2Y_12_ with TfR was determined by image segmentation using the ImageJ plugin SQUASSH (Rizk et al., 2014).

### Live-cell TIRF imaging and data analysis

For live-cell imaging, cells were transfected as described above and split into an eight-well microscopy chamber on the day before imaging. Cells were imaged in an environmental controlled chamber at 37°C in the presence of CO_2_-independent media. Cells were imaged using a Zeiss Cell Observer with TIRF3 fitted with an α Plan Apochromat 100× 1.46 NA lens controlled by Axiovision. To reduce phototoxicity, cells were imaged at low laser power of ≤1% in two channels by sequential excitation at 1 Hz and were detected with a charge-coupled device camera with a pixel size of 16 µm. The depth of the evanescent field was 60–70 nm. For arrestin3 clustering, 1321N1 cells were serum starved for 1 h in the presence of 0.1 U/ml apyrase and imaged directly before and after addition of 10 mM ADP into the imaging media. Lifetime dynamics of CCPs and recruitment of adaptor proteins was determined using the MATLAB script cmeAnalysis (Aguet et al., 2013). Multiple cells from different repeats were pooled to account for cell to cell variability, and outliers were removed according to the criteria of the program.

### FRAP imaging

For FRAP measurements, 1321N1 cells were transfected with CLCb^WT^-GFP or CLCb^S204A^-GFP as described above, while HeLa cells stably expressing CLCb^WT^-GFP or CLCb^SallA^-GFP were treated with 10 mM doxycycline and transferred into Ibidi glass-bottomed 35-mm dishes the day before imaging. For 1321N1 cells, recovery traces were determined without the presence of ADP in serum-free media and again directly after addition of 10 mM ADP within a 10-min window. Cells were imaged in an environmentally controlled chamber at 37°C in the presence of CO_2_-independent media using a Nikon A1 confocal system with a CFI Plan Apochromat VC 60× 1.4 NA oil lens. An area of 256 × 256 pixels at the plasma membrane of transfected cells was imaged at 0.1-µm pixel size. Single CCSs were identified and imaged for 3 s at 1 Hz before bleaching the GFP signal for 1 s at 100% laser power using the 403 nm and 488 nm laser lines. Recovery of the signal was recorded over 30 s at 1 Hz at laser powers <1% to reduce photobleaching. Recovery traces were determined in two regions of interest (ROIs) of 7 × 7 pixels containing a single CCS and a control ROI devoid of CCS to determine the background signal. Background signal was subtracted from each CCS trace. The first three background-corrected frames were averaged and set to 100% for normalization, while the signal from the background ROI at the time of bleaching was set to 0% to account for recovery from out-of-focus GFP signal, unrelated to the signal from the CCS. The background-corrected signal directly after photobleaching was defined as bleaching efficiency and represents *t* = 0 of the recovery trace. Only traces with a bleaching efficiency of ≥80% were used for further analysis. All normalized recovery traces were averaged, the SD was determined, and a hyperbola curve of *y*(*t*) = *offset* + *MF* * *t*/(*t* + *t*_1/2_) was fitted based on the minimal squared difference. The offset was determined as the average bleaching efficiency from all traces, with *t*_1/2_ being the halftime of recovery.

### EM

#### Resin-embedded sections

HeLa cells expressing CLCb^WT^-GFP or CLCb^SallA^-GFP were grown in media containing 10% FCS and embedded in resin according to standard protocols (Smythe et al., 1989). In brief, cells were grown on 60-mm dishes, fixed with 4% PFA, pelleted, and postfixed with 1% glutaraldehyde and 4% tannic acid. Pellets were staining with 1% osmium for 1 h and 0.5% uranyl acetate overnight, embedded in epon resin, cut into 70-nm sections, and stained en bloc with uranyl acetate and lead citrate before imaging using an FEI Tecnai T12 Spirit at 80 kV. Samples were processed blind, and CCPs were analyzed by determining the width of the neck and the depth of each CCP using ImageJ as well as tracing around their contours manually using Adobe Illustrator.

#### Unroofed cells

Adherent plasma membrane from HeLa cells plated on glass coverslips were grown in DMEM containing 10% FCS and disrupted by sonication as described previously (Heuser, 2000). Glutaraldehyde/PFA-fixed cells were further sequentially treated with OsO_4_, tannic acid, and uranyl acetate before dehydration and hexamethyldisilazane drying (Sigma-Aldrich). Dried samples were then rotary shadowed with platinum and carbon with a high-vacuum sputter coater (Leica Microsystems). Platinum replicas were floated off the glass by angled immersion into hydrofluoric acid, washed several times by flotation on distilled water, and picked up on 200 mesh formvar/carbon-coated EM grids. The grids were mounted in a eucentric side-entry goniometer stage of a transmission electron microscope operated at 80 kV (CM120; Philips), and images were recorded with a Morada digital camera (Olympus). Images were processed in Adobe Photoshop to adjust brightness and contrast and presented in inverted contrast.

### Endocytosis assays

Endocytosis of the P2Y_12_ receptor was determined as described by Ferreira et al. (2012). In brief, the extracellular HA tag on the P2Y_12_ receptor was used in an ELISA to determine the surface receptor level after various time points following stimulation with 10 mM ADP. The level of surface receptor without stimulation was set to 100%. Endocytosis of biotinylated transferrin was measured by its loss of accessibility to exogenously added avidin following internalization as previously described (Smythe et al., 1992). Briefly, cells were incubated with 1 µg/ml biotinylated transferrin either in serum-free media following serum starvation for 1 h before the experiment or in media containing 10% FCS without prior serum starvation. At different times, as indicated in figure legends, cells were washed 2× with ice-cold PBS/0.2% BSA and incubated for 30 min with 50 µg/ml avidin dissolved in PBS/0.2% BSA. Cells were then washed and incubated in PBS/0.2% BSA containing 1 mg/ml biocytin for 10 min before solubilization in buffer A (20 mM Tris, pH 7.5, 100 mM NaCl, 1 mM MgCl_2_, 1% Triton X-100, and 0.1% SDS). Lysates were applied to ELISA plates coated with antitransferrin antibody (a gift from the Scottish Antibody Production Unit) and incubated overnight at 4°C. ELISA plates were washed 3× with buffer A and incubated with streptavidin-HRP for 1 h. Following washing, 200 µl HRP substrate, o-phenylenediamine (Sigma-Aldrich; 25 mg in 25 ml assay buffer: 51 mM sodium citrate and 27 mM sodium phosphate, pH 5.0, plus 10 µl H_2_O_2_) was added to each well. The reaction was terminated by addition of 50 µl of 2 M sulphuric acid. Absorbance at 492 nm was read on an ELISA plate reader. The total amount of internalized biotinylated transferrin was determined from samples that were not treated with avidin.

For internalization of fluorescently labeled transferrin, 1321N1 cells were transfected with either CLC^WT^-GFP or CLC^SallA^-GFP using PolyFect and serum starved as well as ATP depleted with 0.1 U/ml apyrase for 1 h. 5 µg/ml transferrin conjugated to Alexa Fluor 568 was added in the presence of 10 mM ADP for the indicated time points and chilled on ice, and then surface-bound transferrin was stripped with acid wash (2 × 5–min washes with 50 mM glycine, 100 mM NaCl, and 2 M urea, pH 3, interspersed with 5-min washes with PBS/0.2% BSA). Coverslips were mounted onto microscopy slides, and whole Z stacks were imaged with a DeltaVision/GE OMX optical microscope with a 60× 1.42 NA oil Plan Apochromat lens in widefield mode with a step size of 400 nm with subsequent deconvolution. Mean fluorescent intensity of internalized transferrin in transfected cells was measured in ImageJ by using the GFP signal as mask on maximum-intensity projections of Z stacks.

### Online supplemental material

Fig. S1 shows cargo influences the properties of CCPs. Fig. S2 shows lifetime dynamics of CCPs. Fig. S3 shows dynamics of eps15, CALM, and epsin2 recruitment to CCP cohorts.

## Supplementary Material

Supplemental Materials (PDF)

## References

[bib1] Aguet F., Antonescu C.N., Mettlen M., Schmid S.L., and Danuser G. 2013 Advances in analysis of low signal-to-noise images link dynamin and AP2 to the functions of an endocytic checkpoint. Dev. Cell. 26:279–291. 10.1016/j.devcel.2013.06.019 23891661 PMC3939604

[bib2] Avinoam O., Schorb M., Beese C.J., Briggs J.A., and Kaksonen M. 2015 Endocytic sites mature by continuous bending and remodeling of the clathrin coat. Science. 348:1369–1372. 10.1126/science.aaa9555 26089517

[bib3] Benmerah A., Bayrou M., Cerf-Bensussan N., and Dautry-Varsat A. 1999 Inhibition of clathrin-coated pit assembly by an Eps15 mutant. J. Cell Sci. 112:1303–1311. 10194409 10.1242/jcs.112.9.1303

[bib4] Bonazzi M., Vasudevan L., Mallet A., Sachse M., Sartori A., Prevost M.C., Roberts A., Taner S.B., Wilbur J.D., Brodsky F.M., and Cossart P. 2011 Clathrin phosphorylation is required for actin recruitment at sites of bacterial adhesion and internalization. J. Cell Biol. 195:525–536. 10.1083/jcb.201105152 22042622 PMC3206339

[bib5] Boucrot E., Pick A., Çamdere G., Liska N., Evergren E., McMahon H.T., and Kozlov M.M. 2012 Membrane fission is promoted by insertion of amphipathic helices and is restricted by crescent BAR domains. Cell. 149:124–136. 10.1016/j.cell.2012.01.047 22464325 PMC3465558

[bib6] Boulant S., Kural C., Zeeh J.C., Ubelmann F., and Kirchhausen T. 2011 Actin dynamics counteract membrane tension during clathrin-mediated endocytosis. Nat. Cell Biol. 13:1124–1131. 10.1038/ncb2307 21841790 PMC3167020

[bib7] Brodsky F.M. 2012 Diversity of clathrin function: new tricks for an old protein. Annu. Rev. Cell Dev. Biol. 28:309–336. 10.1146/annurev-cellbio-101011-155716 22831640

[bib8] Bucher D., Frey F., Sochacki K.A., Kummer S., Bergeest J.P., Godinez W.J., Kräusslich H.G., Rohr K., Taraska J.W., Schwarz U.S., and Boulant S. 2018 Clathrin-adaptor ratio and membrane tension regulate the flat-to-curved transition of the clathrin coat during endocytosis. Nat. Commun. 9:1109 10.1038/s41467-018-03533-0 29549258 PMC5856840

[bib9] Chen H., Fre S., Slepnev V.I., Capua M.R., Takei K., Butler M.H., Di Fiore P.P., and De Camilli P. 1998 Epsin is an EH-domain-binding protein implicated in clathrin-mediated endocytosis. Nature. 394:793–797. 10.1038/29555 9723620

[bib10] Chen P.H., Bendris N., Hsiao Y.J., Reis C.R., Mettlen M., Chen H.Y., Yu S.L., and Schmid S.L. 2017 Crosstalk between CLCb/Dyn1-Mediated Adaptive Clathrin-Mediated Endocytosis and Epidermal Growth Factor Receptor Signaling Increases Metastasis. Dev. Cell. 40:278–288. 28171750 10.1016/j.devcel.2017.01.007 PMC5740869

[bib11] Copic A., Latham C.F., Horlbeck M.A., D’Arcangelo J.G., and Miller E.A. 2012 ER cargo properties specify a requirement for COPII coat rigidity mediated by Sec13p. Science. 335:1359–1362. 10.1126/science.1215909 22300850 PMC3306526

[bib12] Dambournet D., Sochacki K.A., Cheng A.T., Akamatsu M., Taraska J.W., Hockemeyer D., and Drubin D.G. 2018 Genome-edited human stem cells expressing fluorescently labeled endocytic markers allow quantitative analysis of clathrin-mediated endocytosis during differentiation. J. Cell Biol. 217:3301–3311. 10.1083/jcb.201710084 29980624 PMC6123002

[bib13] Dannhauser P.N., and Ungewickell E.J. 2012 Reconstitution of clathrin-coated bud and vesicle formation with minimal components. Nat. Cell Biol. 14:634–639. 10.1038/ncb2478 22522172

[bib14] Dannhauser P.N., Platen M., Böning H., Ungewickell H., Schaap I.A., and Ungewickell E.J. 2015 Effect of clathrin light chains on the stiffness of clathrin lattices and membrane budding. Traffic. 16:519–533. 10.1111/tra.12263 25652138

[bib15] den Otter W.K., and Briels W.J. 2011 The generation of curved clathrin coats from flat plaques. Traffic. 12:1407–1416. 10.1111/j.1600-0854.2011.01241.x 21718403

[bib16] Ehrlich M., Boll W., Van Oijen A., Hariharan R., Chandran K., Nibert M.L., and Kirchhausen T. 2004 Endocytosis by random initiation and stabilization of clathrin-coated pits. Cell. 118:591–605. 10.1016/j.cell.2004.08.017 15339664

[bib17] Eichholtz T., Jalink K., Fahrenfort I., and Moolenaar W.H. 1993 The bioactive phospholipid lysophosphatidic acid is released from activated platelets. Biochem. J. 291:677–680. 10.1042/bj2910677 8489494 PMC1132420

[bib18] Eisenberg E., and Greene L.E. 2007 Multiple roles of auxilin and hsc70 in clathrin-mediated endocytosis. Traffic. 8:640–646. 10.1111/j.1600-0854.2007.00568.x 17488288

[bib19] Ferreira F., Foley M., Cooke A., Cunningham M., Smith G., Woolley R., Henderson G., Kelly E., Mundell S., and Smythe E. 2012 Endocytosis of G protein-coupled receptors is regulated by clathrin light chain phosphorylation. Curr. Biol. 22:1361–1370. 10.1016/j.cub.2012.05.034 22704991

[bib20] Greener T., Grant B., Zhang Y., Wu X., Greene L.E., Hirsh D., and Eisenberg E. 2001 Caenorhabditis elegans auxilin: a J-domain protein essential for clathrin-mediated endocytosis in vivo. Nat. Cell Biol. 3:215–219. 10.1038/35055137 11175756

[bib21] Grove J., Metcalf D.J., Knight A.E., Wavre-Shapton S.T., Sun T., Protonotarios E.D., Griffin L.D., Lippincott-Schwartz J., and Marsh M. 2014 Flat clathrin lattices: stable features of the plasma membrane. Mol. Biol. Cell. 25:3581–3594. 10.1091/mbc.e14-06-1154 25165141 PMC4230618

[bib22] Hanyaloglu A.C., and von Zastrow M. 2008 Regulation of GPCRs by endocytic membrane trafficking and its potential implications. Annu. Rev. Pharmacol. Toxicol. 48:537–568. 10.1146/annurev.pharmtox.48.113006.094830 18184106

[bib23] Henry A.G., Hislop J.N., Grove J., Thorn K., Marsh M., and von Zastrow M. 2012 Regulation of endocytic clathrin dynamics by cargo ubiquitination. Dev. Cell. 23:519–532. 10.1016/j.devcel.2012.08.003 22940114 PMC3470869

[bib24] Heuser J. 2000 The production of ‘cell cortices’ for light and electron microscopy. Traffic. 1:545–552. 10.1034/j.1600-0854.2000.010704.x 11208142

[bib25] Hinrichsen L., Harborth J., Andrees L., Weber K., and Ungewickell E.J. 2003 Effect of clathrin heavy chain- and alpha-adaptin-specific small inhibitory RNAs on endocytic accessory proteins and receptor trafficking in HeLa cells. J. Biol. Chem. 278:45160–45170. 10.1074/jbc.M307290200 12960147

[bib26] Hirst J., Sahlender D.A., Li S., Lubben N.B., Borner G.H., and Robinson M.S. 2008 Auxilin depletion causes self-assembly of clathrin into membraneless cages in vivo. Traffic. 9:1354–1371. 10.1111/j.1600-0854.2008.00764.x 18489706 PMC2628426

[bib27] Holkar S.S., Kamerkar S.C., and Pucadyil T.J. 2015 Spatial Control of Epsin-induced Clathrin Assembly by Membrane Curvature. J. Biol. Chem. 290:14267–14276. 10.1074/jbc.M115.653394 25837255 PMC4505496

[bib28] Hopkins C.R., and Trowbridge I.S. 1983 Internalization and processing of transferrin and the transferrin receptor in human carcinoma A431 cells. J. Cell Biol. 97:508–521. 10.1083/jcb.97.2.508 6309862 PMC2112524

[bib29] Huang F., Khvorova A., Marshall W., and Sorkin A. 2004 Analysis of clathrin-mediated endocytosis of epidermal growth factor receptor by RNA interference. J. Biol. Chem. 279:16657–16661. 10.1074/jbc.C400046200 14985334

[bib30] Irannejad R., and von Zastrow M. 2014 GPCR signaling along the endocytic pathway. Curr. Opin. Cell Biol. 27:109–116. 10.1016/j.ceb.2013.10.003 24680436 PMC4968408

[bib31] Kaksonen M., and Roux A. 2018 Mechanisms of clathrin-mediated endocytosis. Nat. Rev. Mol. Cell Biol. 19:313–326. 10.1038/nrm.2017.132 29410531

[bib32] Kanaseki T., and Kadota K. 1969 The “vesicle in a basket”. A morphological study of the coated vesicle isolated from the nerve endings of the guinea pig brain, with special reference to the mechanism of membrane movements. J. Cell Biol. 42:202–220. 10.1083/jcb.42.1.202 4182372 PMC2107565

[bib33] Kelly E., Bailey C.P., and Henderson G. 2008 Agonist-selective mechanisms of GPCR desensitization. Br. J. Pharmacol. 153(S1, Suppl 1):S379–S388. 10.1038/sj.bjp.0707604 18059321 PMC2268061

[bib34] Keyel P.A., Mishra S.K., Roth R., Heuser J.E., Watkins S.C., and Traub L.M. 2006 A single common portal for clathrin-mediated endocytosis of distinct cargo governed by cargo-selective adaptors. Mol. Biol. Cell. 17:4300–4317. 10.1091/mbc.e06-05-0421 16870701 PMC1635374

[bib35] Kirchhausen T., and Harrison S.C. 1981 Protein organization in clathrin trimers. Cell. 23:755–761. 10.1016/0092-8674(81)90439-6 7226229

[bib36] Kirchhausen T., Owen D., and Harrison S.C. 2014 Molecular structure, function, and dynamics of clathrin-mediated membrane traffic. Cold Spring Harb. Perspect. Biol. 6:a016725 10.1101/cshperspect.a016725 24789820 PMC3996469

[bib37] Lampe M., Pierre F., Al-Sabah S., Krasel C., and Merrifield C.J. 2014 Dual single-scission event analysis of constitutive transferrin receptor (TfR) endocytosis and ligand-triggered β2-adrenergic receptor (β2AR) or Mu-opioid receptor (MOR) endocytosis. Mol. Biol. Cell. 25:3070–3080. 10.1091/mbc.e14-06-1112 25079691 PMC4230595

[bib38] Lampe M., Vassilopoulos S., and Merrifield C. 2016 Clathrin coated pits, plaques and adhesion. J. Struct. Biol. 196:48–56. 10.1016/j.jsb.2016.07.009 27431447

[bib39] Lee D.W., Wu X., Eisenberg E., and Greene L.E. 2006 Recruitment dynamics of GAK and auxilin to clathrin-coated pits during endocytosis. J. Cell Sci. 119:3502–3512. 10.1242/jcs.03092 16895969

[bib40] Leyton-Puig D., Isogai T., Argenzio E., van den Broek B., Klarenbeek J., Janssen H., Jalink K., and Innocenti M. 2017 Flat clathrin lattices are dynamic actin-controlled hubs for clathrin-mediated endocytosis and signalling of specific receptors. Nat. Commun. 8:16068 10.1038/ncomms16068 28703125 PMC5511353

[bib41] Loerke D., Mettlen M., Yarar D., Jaqaman K., Jaqaman H., Danuser G., and Schmid S.L. 2009 Cargo and dynamin regulate clathrin-coated pit maturation. PLoS Biol. 7:e1000057 10.1371/journal.pbio.1000057 PMC2656549 19296720

[bib42] Loerke D., Mettlen M., Schmid S.L., and Danuser G. 2011 Measuring the hierarchy of molecular events during clathrin-mediated endocytosis. Traffic. 12:815–825. 10.1111/j.1600-0854.2011.01197.x 21447041 PMC3115502

[bib43] Ma L., Umasankar P.K., Wrobel A.G., Lymar A., McCoy A.J., Holkar S.S., Jha A., Pradhan-Sundd T., Watkins S.C., Owen D.J., and Traub L.M. 2016 Transient Fcho1/2⋅Eps15/R⋅AP-2 Nanoclusters Prime the AP-2 Clathrin Adaptor for Cargo Binding. Dev. Cell. 37:428–443. 10.1016/j.devcel.2016.05.003 27237791 PMC4921775

[bib44] Maib H., Smythe E., and Ayscough K. 2017 Forty years on: clathrin-coated pits continue to fascinate. Mol. Biol. Cell. 28:843–847. 10.1091/mbc.e16-04-0213 28360213 PMC5385932

[bib45] McMahon H.T., and Boucrot E. 2011 Molecular mechanism and physiological functions of clathrin-mediated endocytosis. Nat. Rev. Mol. Cell Biol. 12:517–533. 10.1038/nrm3151 21779028

[bib46] Merrifield C.J., Feldman M.E., Wan L., and Almers W. 2002 Imaging actin and dynamin recruitment during invagination of single clathrin-coated pits. Nat. Cell Biol. 4:691–698. 10.1038/ncb837 12198492

[bib47] Messa M., Fernández-Busnadiego R., Sun E.W., Chen H., Czapla H., Wrasman K., Wu Y., Ko G., Ross T., Wendland B., and De Camilli P. 2014 Epsin deficiency impairs endocytosis by stalling the actin-dependent invagination of endocytic clathrin-coated pits. eLife. 3:e03311 10.7554/eLife.03311 25122462 PMC4161027

[bib48] Mettlen M., Stoeber M., Loerke D., Antonescu C.N., Danuser G., and Schmid S.L. 2009 Endocytic accessory proteins are functionally distinguished by their differential effects on the maturation of clathrin-coated pits. Mol. Biol. Cell. 20:3251–3260. 10.1091/mbc.e09-03-0256 19458185 PMC2710825

[bib49] Mettlen M., Chen P.H., Srinivasan S., Danuser G., and Schmid S.L. 2018 Regulation of Clathrin-Mediated Endocytosis. Annu. Rev. Biochem. 87:871–896. 10.1146/annurev-biochem-062917-012644 29661000 PMC6383209

[bib50] Meyerholz A., Hinrichsen L., Groos S., Esk P.C., Brandes G., and Ungewickell E.J. 2005 Effect of clathrin assembly lymphoid myeloid leukemia protein depletion on clathrin coat formation. Traffic. 6:1225–1234. 10.1111/j.1600-0854.2005.00355.x 16262731

[bib51] Miller S.E., Mathiasen S., Bright N.A., Pierre F., Kelly B.T., Kladt N., Schauss A., Merrifield C.J., Stamou D., Höning S., and Owen D.J. 2015 CALM regulates clathrin-coated vesicle size and maturation by directly sensing and driving membrane curvature. Dev. Cell. 33:163–175. 10.1016/j.devcel.2015.03.002 25898166 PMC4406947

[bib52] Mundell S.J., Luo J., Benovic J.L., Conley P.B., and Poole A.W. 2006 Distinct clathrin-coated pits sort different G protein-coupled receptor cargo. Traffic. 7:1420–1431. 10.1111/j.1600-0854.2006.00469.x 16899088

[bib53] Newmyer S.L., Christensen A., and Sever S. 2003 Auxilin-dynamin interactions link the uncoating ATPase chaperone machinery with vesicle formation. Dev. Cell. 4:929–940. 10.1016/S1534-5807(03)00157-6 12791276

[bib54] Rizk A., Paul G., Incardona P., Bugarski M., Mansouri M., Niemann A., Ziegler U., Berger P., and Sbalzarini I.F. 2014 Segmentation and quantification of subcellular structures in fluorescence microscopy images using Squassh. Nat. Protoc. 9:586–596. 10.1038/nprot.2014.037 24525752

[bib55] Sahlender D.A., Kozik P., Miller S.E., Peden A.A., and Robinson M.S. 2013 Uncoupling the functions of CALM in VAMP sorting and clathrin-coated pit formation. PLoS One. 8:e64514 10.1371/journal.pone.0064514 23741335 PMC3669311

[bib56] Saleem M., Morlot S., Hohendahl A., Manzi J., Lenz M., and Roux A. 2015 A balance between membrane elasticity and polymerization energy sets the shape of spherical clathrin coats. Nat. Commun. 6:6249 10.1038/ncomms7249 25695735 PMC4346611

[bib57] Schmid S.L., Matsumoto A.K., and Rothman J.E. 1982 A domain of clathrin that forms coats. Proc. Natl. Acad. Sci. USA. 79:91–95. 10.1073/pnas.79.1.91 6948304 PMC345667

[bib58] Schmid S.L., Braell W.A., Schlossman D.M., and Rothman J.E. 1984 A role for clathrin light chains in the recognition of clathrin cages by ‘uncoating ATPase’. Nature. 311:228–231. 10.1038/311228a0 6148701

[bib59] Scott B.L., Sochacki K.A., Low-Nam S.T., Bailey E.M., Luu Q., Hor A., Dickey A.M., Smith S., Kerkvliet J.G., Taraska J.W., and Hoppe A.D. 2018 Membrane bending occurs at all stages of clathrin-coat assembly and defines endocytic dynamics. Nat. Commun. 9:419 10.1038/s41467-018-02818-8 29379015 PMC5789089

[bib60] Smythe E., Pypaert M., Lucocq J., and Warren G. 1989 Formation of coated vesicles from coated pits in broken A431 cells. J. Cell Biol. 108:843–853. 10.1083/jcb.108.3.843 2564003 PMC2115393

[bib61] Smythe E., Carter L.L., and Schmid S.L. 1992 Cytosol- and clathrin-dependent stimulation of endocytosis in vitro by purified adaptors. J. Cell Biol. 119:1163–1171. 10.1083/jcb.119.5.1163 1447294 PMC2289721

[bib62] Smythe E., Smith P.D., Jacob S.M., Theobald J., and Moss S.E. 1994 Endocytosis occurs independently of annexin VI in human A431 cells. J. Cell Biol. 124:301–306. 10.1083/jcb.124.3.301 7905003 PMC2119942

[bib63] Soohoo A.L., and Puthenveedu M.A. 2013 Divergent modes for cargo-mediated control of clathrin-coated pit dynamics. Mol. Biol. Cell. 24:1725–1734. 23536704 10.1091/mbc.E12-07-0550 PMC3667725

[bib64] Sorokin A., Heil K.F., Armstrong J.D., and Sorokina O. 2018 Rule-based modelling provides an extendable framework for comparing candidate mechanisms underpinning clathrin polymerisation. Sci. Rep. 8:5658 10.1038/s41598-018-23829-x 29618727 PMC5884807

[bib65] Stachowiak J.C., Brodsky F.M., and Miller E.A. 2013 A cost-benefit analysis of the physical mechanisms of membrane curvature. Nat. Cell Biol. 15:1019–1027. 10.1038/ncb2832 23999615 PMC3813008

[bib66] Taylor M.J., Perrais D., and Merrifield C.J. 2011 A high precision survey of the molecular dynamics of mammalian clathrin-mediated endocytosis. PLoS Biol. 9:e1000604 10.1371/journal.pbio.1000604 21445324 PMC3062526

[bib67] Taylor M.J., Lampe M., and Merrifield C.J. 2012 A feedback loop between dynamin and actin recruitment during clathrin-mediated endocytosis. PLoS Biol. 10:e1001302 10.1371/journal.pbio.1001302 22505844 PMC3323523

[bib68] Tebar F., Bohlander S.K., and Sorkin A. 1999 Clathrin assembly lymphoid myeloid leukemia (CALM) protein: localization in endocytic-coated pits, interactions with clathrin, and the impact of overexpression on clathrin-mediated traffic. Mol. Biol. Cell. 10:2687–2702. 10.1091/mbc.10.8.2687 10436022 PMC25500

[bib69] Ungewickell E. 1983 Biochemical and immunological studies on clathrin light chains and their binding sites on clathrin triskelions. EMBO J. 2:1401–1408. 10.1002/j.1460-2075.1983.tb01598.x 10872337 PMC555289

[bib70] Ungewickell E., Ungewickell H., Holstein S.E., Lindner R., Prasad K., Barouch W., Martin B., Greene L.E., and Eisenberg E. 1995 Role of auxilin in uncoating clathrin-coated vesicles. Nature. 378:632–635. 10.1038/378632a0 8524399

[bib71] Watts C. 1985 Rapid endocytosis of the transferrin receptor in the absence of bound transferrin. J. Cell Biol. 100:633–637. 10.1083/jcb.100.2.633 2857182 PMC2113434

[bib72] Wilbur J.D., Hwang P.K., Ybe J.A., Lane M., Sellers B.D., Jacobson M.P., Fletterick R.J., and Brodsky F.M. 2010 Conformation switching of clathrin light chain regulates clathrin lattice assembly. Dev. Cell. 18:854–861. 10.1016/j.devcel.2010.04.007 PMC2975025 20493816

[bib73] Wu S., Majeed S.R., Evans T.M., Camus M.D., Wong N.M., Schollmeier Y., Park M., Muppidi J.R., Reboldi A., Parham P., 2016 Clathrin light chains’ role in selective endocytosis influences antibody isotype switching. Proc. Natl. Acad. Sci. USA. 113:9816–9821. 10.1073/pnas.1611189113 27540116 PMC5024586

[bib74] Wu X., Zhao X., Baylor L., Kaushal S., Eisenberg E., and Greene L.E. 2001 Clathrin exchange during clathrin-mediated endocytosis. J. Cell Biol. 155:291–300. 10.1083/jcb.200104085 11604424 PMC2198830

[bib75] Ybe J.A., Greene B., Liu S.H., Pley U., Parham P., and Brodsky F.M. 1998 Clathrin self-assembly is regulated by three light-chain residues controlling the formation of critical salt bridges. EMBO J. 17:1297–1303. 10.1093/emboj/17.5.1297 9482727 PMC1170478

[bib76] Ybe J.A., Perez-Miller S., Niu Q., Coates D.A., Drazer M.W., and Clegg M.E. 2007 Light chain C-terminal region reinforces the stability of clathrin heavy chain trimers. Traffic. 8:1101–1110. 10.1111/j.1600-0854.2007.00597.x 17555534

[bib77] Yim Y.I., Scarselletta S., Zang F., Wu X., Lee D.W., Kang Y.S., Eisenberg E., and Greene L.E. 2005 Exchange of clathrin, AP2 and epsin on clathrin-coated pits in permeabilized tissue culture cells. J. Cell Sci. 118:2405–2413. 10.1242/jcs.02356 15923653

[bib78] Yim Y.I., Sun T., Wu L.G., Raimondi A., De Camilli P., Eisenberg E., and Greene L.E. 2010 Endocytosis and clathrin-uncoating defects at synapses of auxilin knockout mice. Proc. Natl. Acad. Sci. USA. 107:4412–4417. 10.1073/pnas.1000738107 20160091 PMC2840126

[bib79] Young A., Stoilova-McPhie S., Rothnie A., Vallis Y., Harvey-Smith P., Ranson N., Kent H., Brodsky F.M., Pearse B.M., Roseman A., and Smith C.J. 2013 Hsc70-induced changes in clathrin-auxilin cage structure suggest a role for clathrin light chains in cage disassembly. Traffic. 14:987–996. 10.1111/tra.12085 23710728 PMC3776051

[bib80] Zhang C.X., Engqvist-Goldstein A.E., Carreno S., Owen D.J., Smythe E., and Drubin D.G. 2005 Multiple roles for cyclin G-associated kinase in clathrin-mediated sorting events. Traffic. 6:1103–1113. 10.1111/j.1600-0854.2005.00346.x 16262722

