# Supplementary material for: Cargo regulates clathrin-coated pit invagination via clathrin light chain phosphorylation
Source: J Cell Biol. 2018 Dec 3;217(12):4253–66. doi: 10.1083/jcb.201805005 (PMC6279376; doi:10.1083/jcb.201805005)
Supplement: Supplemental Materials (PDF) [file JCB_201805005_sm.pdf]

Supplemental material

Maib et al., https://doi.org/10.1083/jcb.201805005

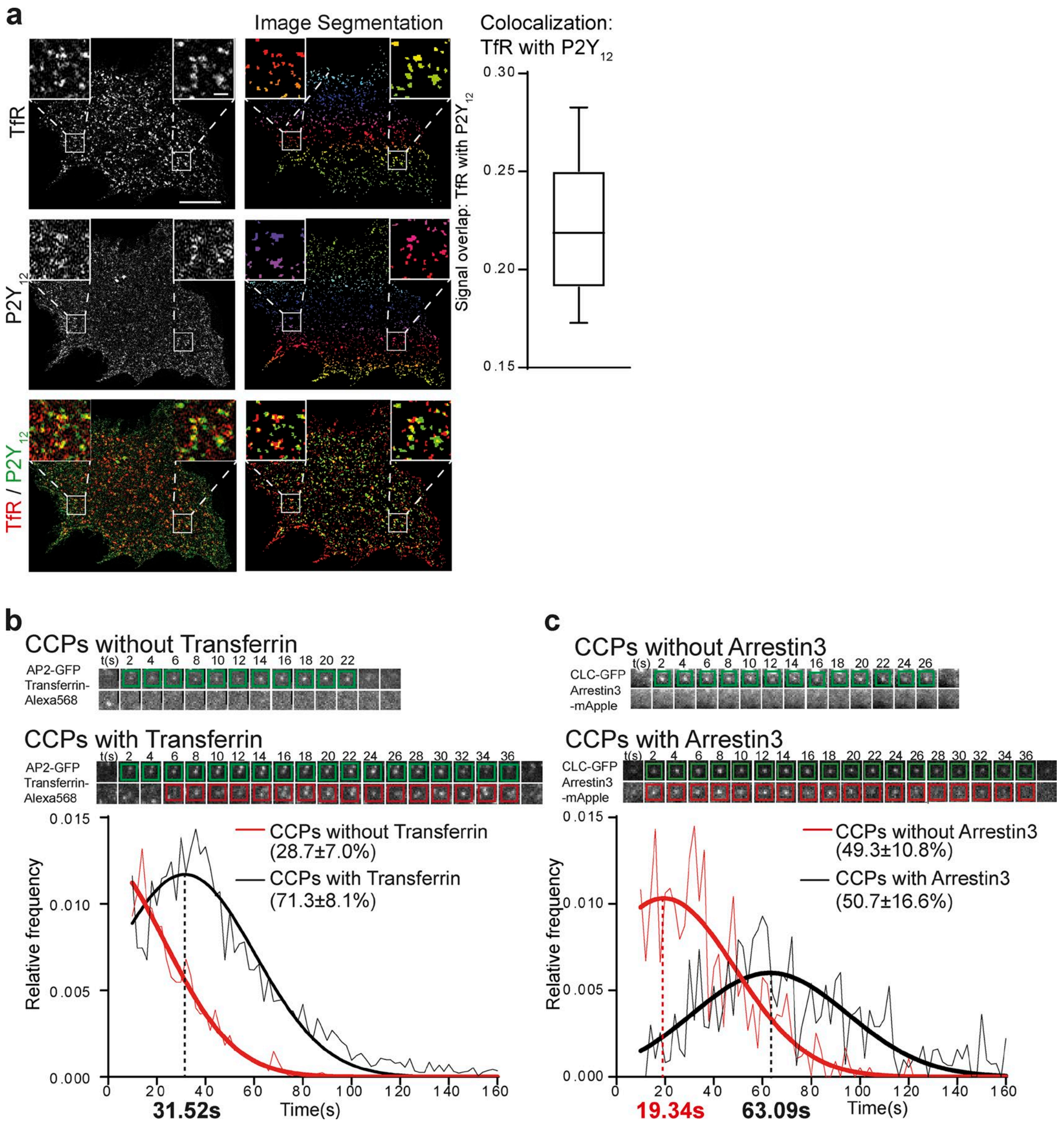

Figure S1. **Cargo packing influences the properties of CCPs. (a)** Colocalization at the plasma membrane of $P2Y_{12}$ with TfR after 3 min ligand stimulation. Images were quantified using the image segmentation tool SQUASSH. n = 22 cells. Box and whiskers plot with 25th to 75th percentiles and range. Bars: 10 µm (main images); 1 µm (insets). **(b)** 1321N1 cells were transfected with AP2-GFP, and the lifetime of CCPs was determined in the presence of Alexa Fluor 568–labeled transferrin and binned for traces with and without detection of transferrin using cmeAnalysis script (Aguet et al., 2013). n = 18,700 traces from 32 cells from three independent repeats. Mean lifetime of CCPs was determined by Gaussian fits. The dataset was reused in Fig. S2 a (left). **(c)** 1321N1 cells were transfected with $CLCb^{WT}$-GFP together with arrestin3-mApple, and the lifetime of CCPs was determined and binned for traces with and without detection of arrestin3 directly after 10 mM ADP stimulation. n = 3,040 traces from 18 cells from three independent experiments. Mean lifetime of CCPs was determined by Gaussian fits. The dataset was reused in Fig. S2 c (left).

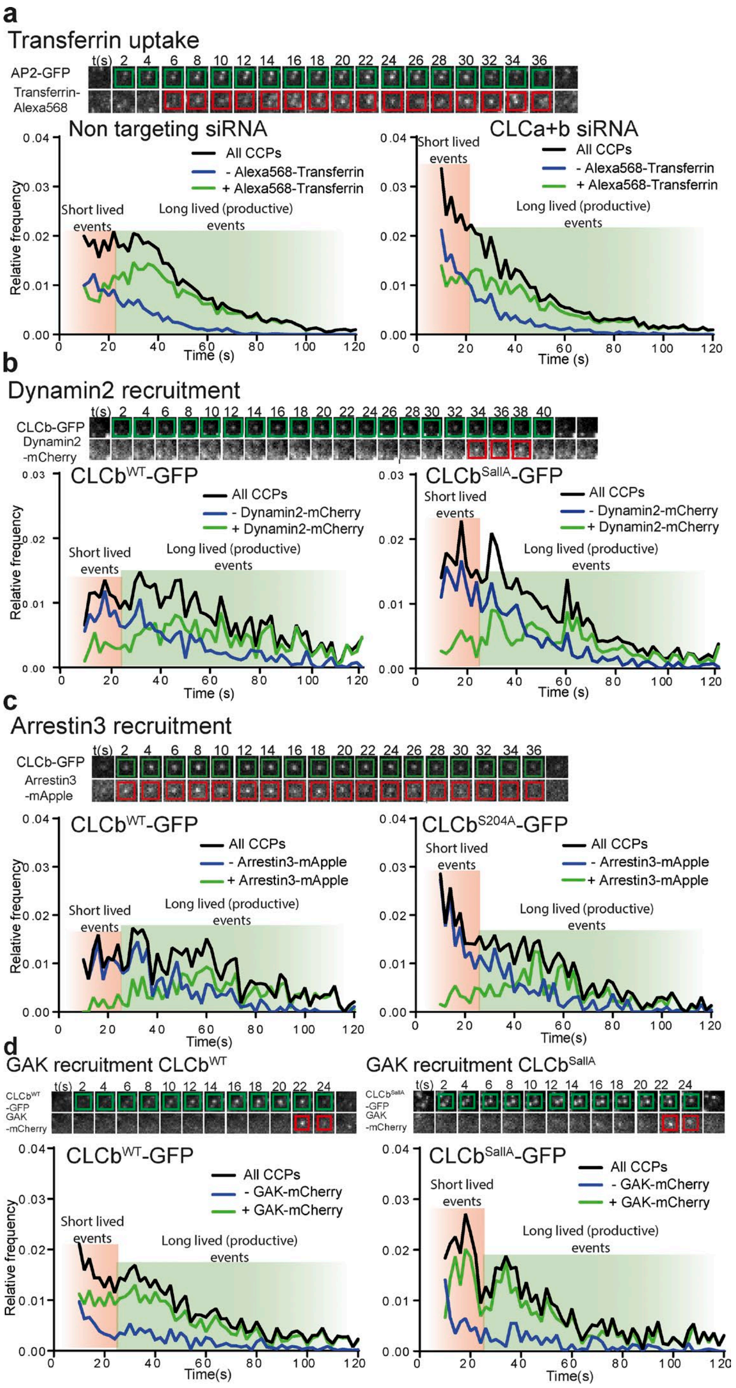

Figure S2. **Lifetime dynamics of CCPs.** **(a)** 1321N1 cells stably expressing HA-tagged $P2Y_{12}$ receptor were treated either with NT control siRNA or siRNA targeting both CLCa and CLCb and then transfected with AP2-GFP, and the lifetime of CCPs was determined in the presence of Alexa Fluor 568–transferrin and binned for traces with (+Alexa-568transferrin, NT siRNA = 71.3 ± 8.1%; CLCa+b siRNA = 65.1 ± 6.6%) and without (−Alexa568-transferrin, NT siRNA = 28.7 ± 7%; CLCa+b siRNA = 34.9 ± 6.8%) detection of transferrin using cmeAnalysis script (Aguet et al., 2013). n > 18,700–25,139 traces from 32 cells each from three independent experiments. The left panel reuses the dataset from Fig. S1 c. **(b)** HeLa cells stably expressing either $CLCb^{WT}$-GFP or $CLCb^{SallA}$-GFP were transfected with dynamin2-mCherry, and the lifetime of CCPs was measured and binned for traces with (+dynamin2-mCherry, $CLCb^{WT}$-GFP = 59.9 ± 11.7%; $CLCb^{SallA}$-GFP = 47.9 ± 13.5%) and without (−dynamin2-mCherry, $CLCb^{WT}$-GFP = 40.1 ± 9.7%; $CLCb^{SallA}$-GFP = 52.1 ± 9.5%) detection of dynamin2. n = 6,721–8,050 traces from 21 cells each from three independent experiments. **(c)** 1321N1 cells were transfected with either $CLCb^{WT}$-GFP or $CLCb^{S204A}$-GFP together with arrestin3-mApple, and the lifetime of CCPs was determined and binned for traces with (+arrestin3-mApple, $CLCb^{WT}$-GFP = 50.7 ± 16.6%; $CLCb^{S204A}$-GFP = 44.8 ± 14.6%) and without (−arrestin3-mApple, $CLCb^{WT}$-GFP = 49.3 ± 10.8%; $CLCb^{S204A}$-GFP = 55.2 ± 14.6%) detection of arrestin3 directly after 10 mM ADP stimulation. n = 3,040–3,069 traces from 18–24 cells from three independent experiments. The left panel reuses the dataset from Fig. S1 d. **(d)** HeLa cells expressing either $CLCb^{WT}$-GFP or $CLCb^{SallA}$-GFP were transfected with GAK-mCherry and imaged by TIRF microscopy, and detection of productive CCPs and GAK recruitment was determined using cmeAnalysis script (Aguet et al., 2013). Lifetime of CCPs was binned for traces with (+GAK-mCherry, $CLCb^{WT}$-GFP = 74.6 ± 12.9%; $CLCb^{SallA}$-GFP = 76.5 ± 12.9%) and without (−GAK-mCherry, $CLCb^{WT}$-GFP = 25.4 ± 8.3%; $CLCb^{SallA}$-GFP = 23.5 ± 7.9%) detection of GAK. n = 5,438–8,379 traces from 20–27 cells from three independent experiments.

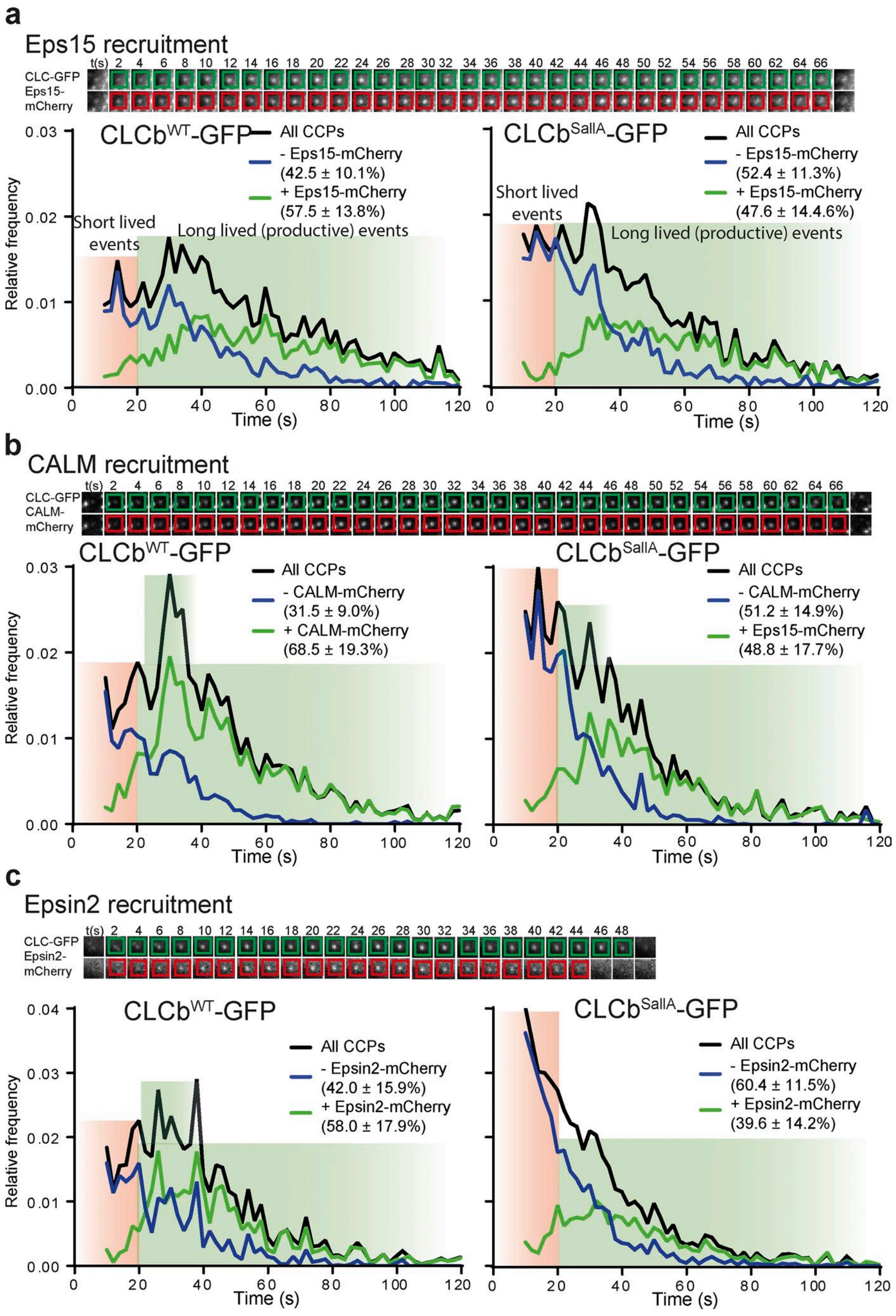

Figure S3. **Dynamics of eps15, CALM, and epsin2 recruitment to CCP cohorts. (a)** HeLa cells stably expressing either $CLCb^{WT}$-GFP or $CLCb^{SallA}$-GFP were transfected with eps15-mCherry, and the lifetime of CCPs was measured and binned for traces with (+eps15-mCherry) and without (−eps15-mCherry) detection of eps15. *n* = 9,397–11,848 traces from 31–34 cells from three independent experiments. **(b)** HeLa cells stably expressing either $CLCb^{WT}$-GFP or $CLCb^{SallA}$-GFP were transfected with CALM-mCherry, and the lifetime of CCPs was measured and binned for traces with (+CALM-mCherry) and without (−CALM-mCherry) detection of CALM. *n* = 7,211–8,605 traces from 29–31 cells from three independent experiments. **(c)** HeLa cells stably expressing either $CLCb^{WT}$-GFP or $CLCb^{SallA}$-GFP were transfected with epsin2-mCherry, and the lifetime of CCPs were measured and binned for traces with (+epsin2-mCherry) and without (−epsin2-mCherry) detection of epsin2. *n* = 5,656–7,351 traces from 22–24 cells from three independent experiments. Percentages refer to the traces in each binned cohort. Lifetimes were measured using cmeAnalysis script (Aguet et al., 2013).

Reference

Aguet, F., C.N. Antonescu, M. Mettlen, S.L. Schmid, and G. Danuser. 2013. Advances in analysis of low signal-to-noise images link dynamin and AP2 to the functions of an endocytic checkpoint. *Dev. Cell.* 26:279–291. https://doi.org/10.1016/j.devcel.2013.06.019
